# Perinatal hypoxia-mediated neurodevelopment abnormalities in congenital heart disease mouse model

**DOI:** 10.1186/s10020-025-01158-w

**Published:** 2025-03-21

**Authors:** Renwei Chen, Haifan Wang, Liqin Zeng, Jiafei He, Xiaohan Liu, Xinting Ji, Paul Yao, Shuo Gu

**Affiliations:** 1 The First Affiliated Hospital, The First Clinical College, Key Laboratory of Emergency and Trauma of Ministry of Education, Hainan Medical University, Haikou, 571199 China; 2 Hainan Women and Children’s Medical Center, Hainan Medical University, Haikou, 570206 China; 3 Department of Gynecology, Sun Yat-Sen University Affiliated No. 8 Hospital, Shenzhen, 518033 China

**Keywords:** Amygdala, Estrogen receptor β, Congenital heart disease, Hypoxia, Neurodevelopment abnormalities

## Abstract

**Background:**

Cyanotic congenital heart disease (CHD) in children has been associated with neurodevelopmental abnormalities, although the underlying mechanisms remain largely unknown. Multiple factors are likely involved in this process. This research aims to explore the potential effects of hypoxia and vascular system-derived factors in neurodevelopmental outcomes in offspring.

**Methods:**

Mouse aorta endothelial cells (MEC) and amygdala neurons were isolated to investigate the effects of hypoxia on pro-inflammatory cytokine release, gene expression, redox balance, mitochondrial function, and epigenetic modifications. A CHD mouse model was established to evaluate the impact of perinatal hypoxia on fetal brain development. Estrogen receptor β (ERβ) expression in endothelial cells was modulated using Tie2-driven lentivirus both in vitro and in vivo study to assess the vascular system’s contribution to hypoxia-mediated neurodevelopmental abnormalities.

**Results:**

Hypoxia exposure, along with factors released from MEC, led to altered gene expression, oxidative stress, mitochondrial dysfunction, and epigenetic modifications in amygdala neurons. In the CHD mouse model, perinatal hypoxia resulted in compromised vascular function, altered gene expression, disrupted redox balance in brain tissues, and impaired behavioral outcomes in offspring. Prenatal expression of ERβ in endothelial cells partially ameliorated these neurodevelopmental abnormalities, while prenatal knockdown of ERβ mimicked the effects of perinatal hypoxia.

**Conclusions:**

Hypoxia, combined with endothelial cell-derived factors, induces epigenetic changes in neurons. In the CHD mouse model, perinatal hypoxia causes vascular dysfunction, altered gene expression, and redox imbalance in brain tissues, leading to behavioral impairments in offspring. Prenatal expression of ERβ in endothelial cells mitigates these effects, suggesting that modulating gene expression in the vascular system during pregnancy could play a protective role against hypoxia-induced neurodevelopmental abnormalities in CHD.

**Supplementary Information:**

The online version contains supplementary material available at 10.1186/s10020-025-01158-w.

## Introduction

Hypoxia, a condition characterized by reduced oxygen supply to tissues, profoundly affects neurodevelopment, particularly in the fetal and neonatal brain. Oxygen deprivation disrupts cellular metabolism, leading to neuronal injury, inflammation, and impaired brain maturation (Palanisamy et al. 2020; Geva et al. 2024). These disruptions can contribute to brain damages, including cognitive deficits, motor dysfunction, and behavioral disorders. Conditions such as perinatal asphyxia, preterm birth, and congenital heart disease (CHD) further elevate the risk of hypoxia-induced neurodevelopmental impairments (Morton et al. 2017; Knirsch et al. 2017). This study explores the role and underlying mechanisms of CHD-mediated neurodevelopmental abnormalities.

CHD is linked to impaired fetal brain development, characterized by smaller brain size, reduced complexity of cortical gyrification, disorganization of white matter tracts, and underdeveloped electrical and biochemical activity, though the underlying mechanism is still unclear (Peyvandi and Rollins 2023; Sha et al. 2023). Additionally, vascular diseases can impair brain function through the heart-brain axis, with numerous factors in CHD potentially contributing directly or indirectly to fetal brain development. These factors may include genetic/epigenetic influences (Chen et al. 2019; Ducsay et al. 2018), cardiovascular abnormalities (Peyvandi and Rollins 2023), and environmental factors (Doehner et al. 2023; Schmitt et al. 2014; Piesova and Mach 2020).

Hypoxic conditions in endothelial cells induce the release of pro-inflammatory factors (Bartels et al. 2013), including monocyte chemoattractant protein-1 (MCP1), tumor necrosis factor α (TNFα) (Li et al. 2024; Ma et al. 2024), interleukin 6 (IL6) and IL1β (Bosco et al. 2004). This also activates the hypoxia-inducible factor 1α (HIF1α) signaling pathway, leading to the upregulation of endothelial nitric oxide synthase (eNOS), vascular endothelial growth factor (VEGF) and stromal cell-derived factor-1 (SDF1) (Ceradini et al. 2008). These released factors may cross the placenta, directly or indirectly interact with neurons, triggering epigenetic changes (Laird et al. 2024; Perez-Perri et al. 2011) and oxidative stress (Yan et al. 2024) during fetal development, ultimately altering gene expression and leading to neurodevelopmental abnormalities (Schmitt et al. 2014).

Estrogen receptor β (ERβ) has been reported to regulate the baseline expression of estrogen-related receptor α (ERRα) (Li et al. 2015) and superoxide dismutase 2 (SOD2) (Liu et al. 2014), thereby influencing redox balance and mitochondrial function in endothelial cells (Kong et al. 2016). Additionally, endothelium-specific ERβ expression via Tie2-driven ERβ lentivirus infection has been shown to alleviate ischemia/reperfusion-induced oxidative stress and vascular damage (Zhan et al. 2016). In this study, Tie2-driven ERβ expression is employed to assess its potential protective effects against perinatal hypoxia-induced neurodevelopmental abnormalities in offspring.

This study hypothesizes that perinatal hypoxia induced by congenital heart disease (CHD) contributes to neurodevelopmental abnormalities in offspring through the release of hypoxia-induced vascular factors, with potential modulation by endothelial ERβ expression. The primary aim is to investigate the underlying mechanisms of these abnormalities, focusing on the effects of hypoxia on vascular factor release and its impact on brain development. By exploring the role of ERβ expression in these processes, the study aims to uncover potential protective mechanisms that may mitigate the impact of perinatal hypoxia on brain development (Xie et al. 2018; Wang et al. 2019; Romanowicz et al. 2021; Zeng et al. 2022).

## Materials and methods

An extended Materials and Methods section can be found in supplementary file Data S1 and primers are listed in Table S1.

### Reagents and materials

Antibodies for eNOS (sc-654), HIF1α (sc-10790), SDF1 (sc-28876), and VEGF (sc-507) were purchased from Santa Cruz Biotech, and antibodies for H3K27me2 (ab24684) and H3K27me3 (ab6002) were obtained from Abcam. Hypoxia was induced by incubating the cells in a hypoxic chamber for 18 h, where oxygen was displaced by flushing with a gas mixture of 95% CO_2_ and 5% N2 for 15 min.

### Methods and rationale

The study employs a maternal CHD model, specifically using MEC to examine hypoxia-induced release of pro-inflammatory cytokines. Additionally, amygdala neurons are utilized to explore the impact of hypoxia on epigenetic modifications, gene expression, redox balance, and mitochondrial function (Xie et al. 2018; Wang et al. 2019). This approach allows for a comprehensive evaluation of how factors released by hypoxia-exposed endothelial cells may affect neuronal function and development. To replicate the conditions seen in CHD-affected neurons, the study incorporates a novel design where neurons are incubated under hypoxic conditions alongside factors released from hypoxia-treated MEC. These factors are hypothesized to cross the placenta, influencing the developing brain both directly and indirectly. A CHD mouse model was established by inducing hypoxia in pregnant dams on embryonic day 16 (E16), with subsequent assessments conducted at multiple time points to evaluate vascular function, redox balance, cytokine levels, and behavioral outcomes in the offspring (Romanowicz et al. 2021). This multi-faceted approach, involving in vitro endothelial cell models and in vivo analysis in a mouse model, was chosen to better understand the interaction between vascular dysfunction (Zeng et al. 2022) and neurodevelopment under hypoxic conditions.

### In vitro cell culture experiments

**Fig. 1:**
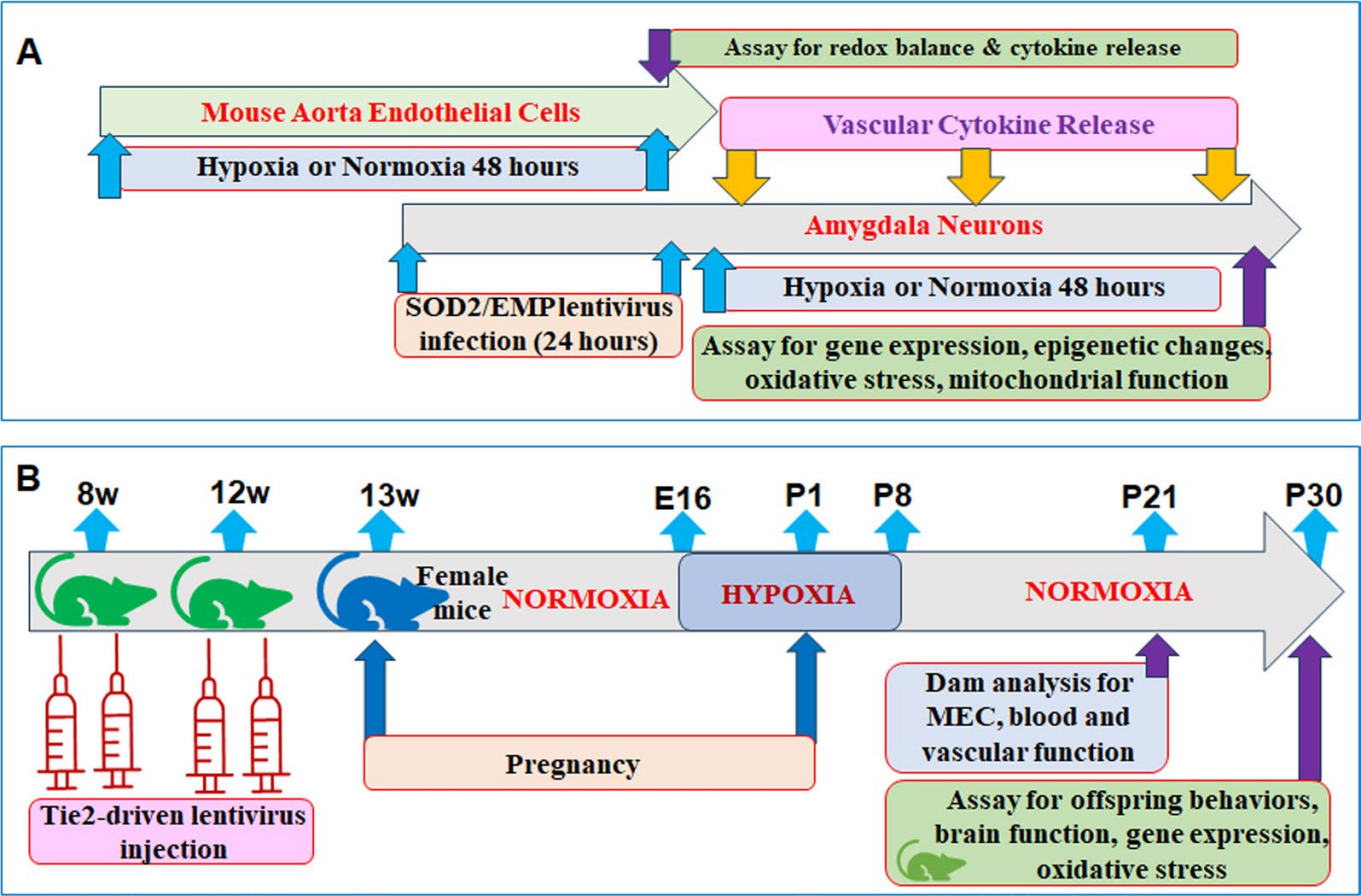
Schematic model for both in vitro and in vivo study. **a** Schematic model for in vitro cell culture study. **b** Schematic model for in vivo mouse study. *E* embryonic, *EMP* empty lentivirus vector, *MEC* mouse aorta endothelial cells, *P* postnatal, *W* weeks, *SOD2* superoxide dismutase 2

Mouse aortic endothelial cells (MEC) were isolated and exposed to either normoxia or hypoxia for 48 h. Hypoxic conditions were induced using a hypoxic chamber, where oxygen was removed by flushing with 95% CO₂ and 5% N₂ for 15 min. Following incubation after 48 h, MEC and supernatants were collected for analysis of gene expression, oxidative stress, and cytokine release, including IL-1β, IL-6, TNF-α, MCP-1, and SDF-1. Amygdala neurons were isolated from embryonic day 18 (E18) mouse embryos and infected with either SOD2 lentivirus (↑SOD2) or an empty control vector (EMP) for 24 h. These neurons were then incubated with 50% supernatant from either hypoxia-treated or normoxia-treated (CTL) MEC under normoxic (NO) or hypoxic (HO) conditions for 48 h. Cells were subsequently collected for analysis of gene expression, epigenetic modifications, redox imbalance, and mitochondrial function (see details Fig. 1a).

### Isolation of mouse aorta endothelial cells (MEC)

The aorta was cleaned with PBS, then placed in ice-cold serum-free DMEM. After opening and rinsing with serum-free DMEM, the aorta was placed intimal-side down on a sterile plate with 0.2% collagenase type I and cultured at 37 °C for 30 min. MEC were scraped, rinsed with DMEM plus serum to stop digestion, and centrifuged. The cell pellet was washed, resuspended in DMEM with antibiotics and endothelial supplements, and cultured in a 60-mm dish, with medium changes every other day. The cells were verified by staining of von Willebrand factor and used at passages P3-P5 (Takeshita et al. 2007; Bodnar et al. 1998).

### In vitro primary culture of amygdala neurons

Primary cultures of amygdala neurons were established by dissecting tissues from untreated mice at embryonic day 18 (E18). The tissues were incubated in 0.05% trypsin EDTA at 37 °C for 15 min, followed by the addition of soybean trypsin inhibitor (Sigma) for 5 min at 37 °C to halt the enzymatic activity. The reaction was then terminated with supplemented Neurobasal A (Invitrogen), and the tissue was mechanically dissociated. The dissociated cells were resuspended in culture medium containing Neurobasal A, B27, 1× GlutaMAX, and 100 U/ml Pen/Strep (Invitrogen), and cultured at 37 °C with 5% CO_2_. The amygdala neurons were then used for subsequent experimental assays (Wang et al. 2019).

### Isolation of mouse PBMC

Heparinized blood was collected via heart puncture, diluted with HBSS, layered onto Ficoll-Paque, and centrifuged. The PBMC layer was collected, washed, and treated with lysing buffer. The leftover cells were washed with HBSS and resuspended for further analysis.

### RT reaction and real-time quantitative PCR

Total RNA was isolated from treated cells utilizing the RNeasy Micro Kit (Qiagen), followed by reverse transcription with the Omniscript RT Kit (Qiagen). Primers were designed via Primer3 Plus software, ensuring a melting temperature of 60 °C, a length of 21 bp, and amplicon sizes ranging between 140 and 160 bp (see Table S1). Primer efficiency was assessed, yielding values between 1.9 and 2.1, and successful amplification was verified using agarose gel electrophoresis. Quantitative real-time PCR was conducted on the iCycler iQ system (Bio-Rad) using the Quantitect SYBR Green PCR Kit (Qiagen). The thermal cycling protocol consisted of an initial denaturation at 95 °C for 8 min, followed by 45 cycles of denaturation at 95 °C, annealing at 60 °C, and extension at 72 °C for 10 s each. A 1 µl aliquot of each cDNA sample was used for target gene quantification. β-actin served as the reference gene for normalization, and relative transcript levels were determined using the ΔΔCT method based on Qiagen’s guidelines. Briefly, the expression levels were analyzed through the comparative threshold cycle approach, normalizing against β-actin. Fold changes in gene expression were calculated as 2^−ΔΔCT^, where CT represents the threshold cycle, ΔCT = CT (target gene) − CT (β-actin), and ΔΔCT = ΔCT (treated sample)  − ΔCT (control sample).

### Chromatin immunoprecipitation (ChIP)

The cells were crosslinked by 1% formaldehyde, quenched with glycine, and lysed. The lysates were sonicated, pre-cleared, and immunoprecipitated with antibodies and Protein A Agarose beads. After washing, elution, and crosslink reversal with Proteinase K, DNA fragments were purified and analyzed by real-time PCR with primers targeting a ~ 150 bp promoter fragment (see primers in Table S1).

### Immunostaining

Cells on gelatin-coated coverslips were fixed with formaldehyde, permeabilized with BSA and Triton X-100, and incubated with 40 μg/ml (dilute 1:50) of primary antibodies (8-oxo-dG or SOD2). After washing, cells were stained with FITC or Texas Red secondary antibodies (1:500) and visualized. Cell nuclei were stained with DAPI, and staining intensity was quantified using Image J.

### DNA methylation analysis

DNA was extracted, bisulfite-modified, and the modified DNA on the ERβ promoter was amplified with the below primers: Methylated primers: forward 5ʹ-aat ttc ggt gtt att att cga aat c-3ʹ, reverse 5ʹ-aaa ttc taa act taa ccg cgt acg-3ʹ; Unmethylated primers: forward 5ʹ-ttt tgg tgt tat tat ttg aaa ttg g-3ʹ, reverse 5ʹ-aat tct aaa ctt aac cac ata cat c-3ʹ. The CpG island size was 108 bp, with Tm values of 64.1 °C for the methylated and 63.3 °C for the unmethylated products. DNA methylation levels were normalized to unmethylated DNA (Ogino et al. 2006; Eads et al. 2000; Nosho et al. 2008).

### Measurement of oxidative stress

Treated cells were seeded in a 96-well plate and incubated with 10 μM CM-H2DCFDA (Invitrogen) for 45 min at 37 °C. Then, the intracellular formation of ROS was measured at excitation/emission wavelengths of 485/530 nm using a FLx800 microplate fluorescence reader (Bio-Tek), and the data was normalized as arbitrary units (Yao et al. 2005). The GSH/GSSG ratio was measured using the GSH/GSSG-Glo™ Assay Kit (#V6611, obtained from Promega) per manufacturers’ instructions. 8-OHdG formation was measured using an OxiSelect™ Oxidative DNA Damage ELISA Kit (#STA320, from Cell Biolabs Inc.) per manufacturers’ instructions and the formation of 8-oxo-dG was determined by immunostaining using primary antibody for 8-oxo-dG (#4354-MC-050, from Novus Biologicals) and quantitated by Image J.

### Measurement of mitochondrial function

Mitochondrial DNA copy numbers were measured by qPCR of ATP6 and β-actin. Intracellular ATP was quantified using a luciferase system. Mitochondrial membrane potential (MMP) was assessed with TMRE staining and quantified by confocal microscopy (Yao et al. 2005; Zou et al. 2017; Zhang et al. 2018, 2017).

### Lentivirus generation

#### SOD2 expression lentivirus

Mouse SOD2 cDNA was cloned into the pLVX-Puro vector using XhoI and XbaI sites. Lentivirus production and cell infection for SOD2 overexpression or empty control were done with the Lenti-X™ System (Clontech) (Zeng et al. 2022).

#### Tie2-driven ERβ expression lentivirus

The Tie2 promoter (1.5 kb upstream and 1st exon) was amplified from mouse DNA and fused with mouse ERβ cDNA. This construct was cloned into the pLVX-Puro vector using *Xho*I and *Xba*I sites. Lentivirus was produced, concentrated, and administered via tail vein injection.

#### Tie2-driven ERβ shRNA lentivirus

The effective shRNA sequence was synthesized, annealed, and fused with the Tie2 promoter using BamHI and *EcoR*I sites. The construct was subcloned into pLVX-shRNA1 vector. Lentivirus was generated for Tie2-shERβ or scrambled control using the Lenti-X™ System (Clontech).

### Perinatal hypoxia mouse model

Three months old female mice were mated, and the confirmed pregnant mice were randomly selected into the below experimental groups: Group 1 (NO-Tie2-EMP): Normoxia with Tie2-driven empty lentivirus injection; Group 2 (HO-Tie2-EMP): Hypoxia with Tie2-driven empty lentivirus injection; Group 3 (HO-Tie2-↑ERβ): Hypoxia with Tie2-driven ERβ expression lentivirus injection; Group 4 (NO-Tie2-shERβ): Normoxia with Tie2-driven ERβ knockdown lentivirus injection. Pregnant mice were placed in a hypoxic chamber beginning on embryonic day 16 (E16). Oxygen concentration in the chamber was continuously maintained, monitored, and recorded at 11 ± 0.5% O₂, with nitrogen gas displacing oxygen. The dams gave birth in hypoxia environment (n = 9), and the pups kept in hypoxia until postnatal day 8 (P8). Control dams (n = 9) were housed and gave birth in normoxia conditions. At P8, the hypoxic mice were returned to normoxia until testing at P30. On embryonic day 21 (E21), after the delivery of pups, dams were euthanized by decapitation at P21 after the weaning process was completed, and blood samples were collected to isolate serum and PBMC. The thoracic aorta was harvested for the isolation of MEC for in vitro assays, and the carotid arteries were collected to determine vascular tension (n = 9). The offspring were studied for behavioral analysis until P30, after which they were euthanized (n = 9). Serum and hematopoietic stem cells (HSC) were collected for vascular function evaluation, and brain tissues were harvested for analyses of oxidative stress and gene expression. Offspring experiments used approximately equal numbers of males and females, with multiple litters contributing to each experimental endpoint (see details in Fig. 1b) (Romanowicz et al. 2021).

### Animal behavior tests

Mouse behaviors were determined using marble-burying test (MBT), elevated plus maze (EPM), open-field test (OFT), novel object recognition (NOR) test, and three-chambered social test.

#### Marble–Burying test (MBT)

The mice were housed in a cage containing 5 cm of bedding, with 20 marbles arranged in a grid layout. After 30 min, a technician counted marbles buried by more than 50% of bedding (Xie et al. 2018; Bahi 2016, 2017; Zou et al. 2017).

#### Elevated plus maze (EPM)

The mice were assessed using an elevated maze consisting of both open and closed arms. Mice were recorded for 5 min to measure time spent in each arm (Zou et al. 2017; Hu et al. 2015).

#### Open-field test (OFT)

Mice were housed in a 40 cm × 40 cm arena for a duration of 30 min. Movement was tracked to assess activity and anxiety, with the central and peripheral zones analyzed (Zou et al. 2017).

#### Novel object recognition (NOR) test

After the OFT, mice explored two identical objects for 5 min. Following a 1-h delay, one object was replaced with a novel one, and exploration times were recorded (Kong et al. 2024).

#### Three-chambered social test

Mice were tested for sociability and social novelty. The test involved exploring chambers with different social and empty cages. Parameters like time spent in each chamber were recorded (Wang et al. 2019; Moy et al. 2004).

### Isolation of brain tissues

Mice were anesthetized, and brain regions (amygdala, hypothalamus, hippocampus) were dissected and stored at − 80 °C for assays (Liu et al. 2020, 2021).

### Preparation of hematopoietic stem Cells (HSC)

The isolation process for HSC was adapted from a previously established method (Rossi et al. 2011). Bone marrow cells were harvested from the tibias of treated mice and stained with specific antibodies to identify HSC (c-Kit+/Sca-1+/Lineage−). To isolate HSC, debris, dead cells, and clumps were removed to ensure only single, viable cells remained. Cells expressing Sca-1 and c-Kit but lacking Lineage markers were then sorted using the BD FACSMelody™ Cell Sorter for further analysis (Xie et al. 2021).

### Monitoring of vascular function

Carotid artery vessel tension was measured using a myograph system. Aortic rings were assessed for vasodilator responses to acetylcholine (Sha et al. 2023; Li et al. 2024).

### Blood pressure measurement

Blood pressure was monitored in conscious mice using telemetry. Mice were implanted with a transmitter, and measurements were taken hourly after a recovery period (Ma et al. 2024; Bosco et al. 2004; Ceradini et al. 2008).

### Immunohistochemistry (IHC)

Amygdala tissue was processed and stained for SOD2. Images were acquired through a confocal microscope and analyzed using Image J (Wang et al. 2019).

## Results

### Hypoxia induces cytokine release and oxidative stress in MEC

**Fig. 2:**
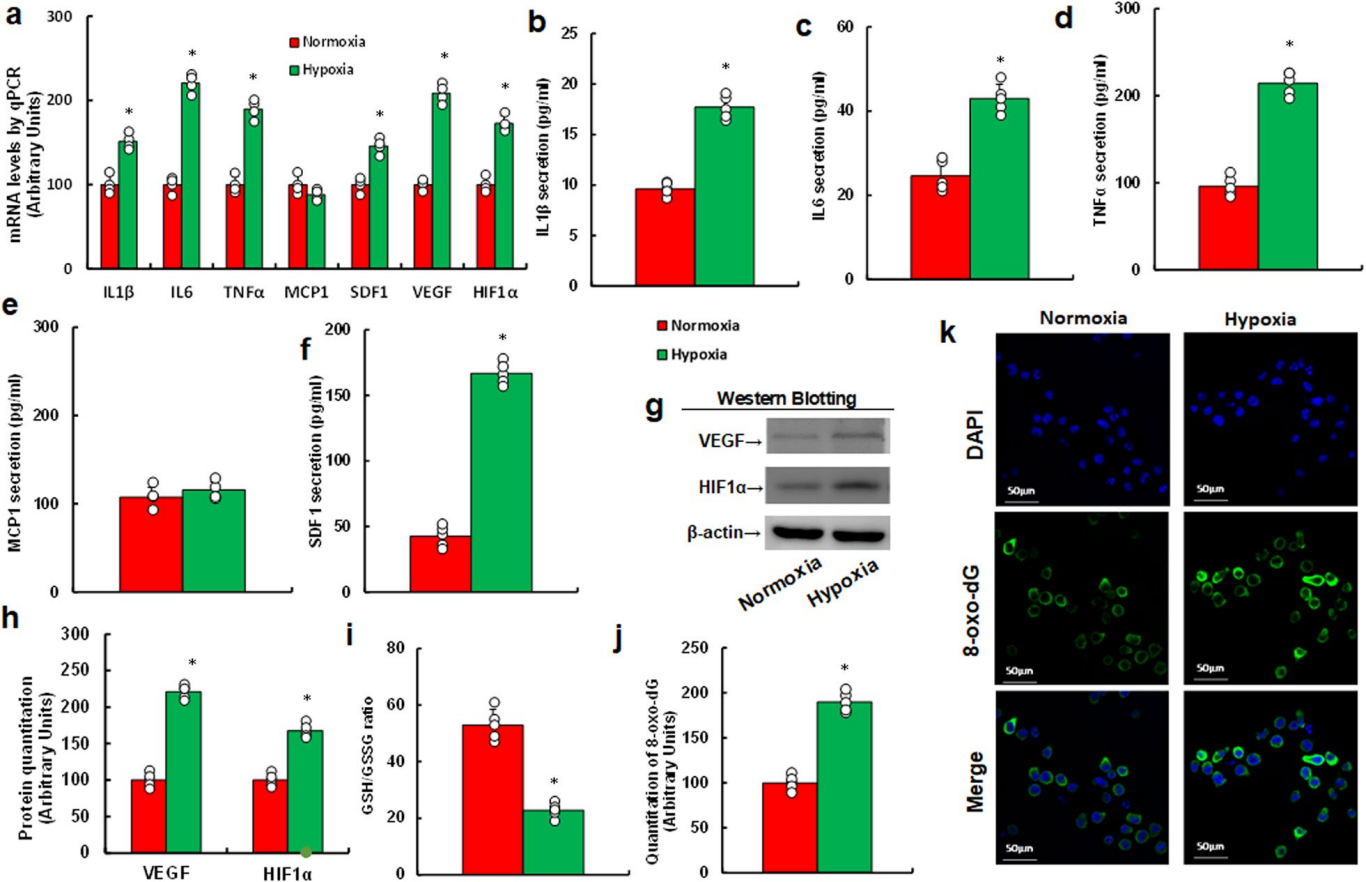
Hypoxia-induced cytokine release and oxidative stress in MEC. Isolated MEC were exposed to either normoxia or hypoxia for 48 h. Cells and supernatants were then collected for analysis. **a** mRNA analysis in MEC, n = 4; **b**–**f** cytokine secretion from supernatants, n = 5: IL1β (**b**), IL6 (**c**), TNFα (**d**), MCP1 (**e**), and SDF1 (**f**); **g** representative western blots; **h** protein quantification for **g**, n = 5; **i** GSH/GSSG ratio in supernatants, n = 5; **j** 8-oxo-dG formation, n = 5; **k** representative images for **j**. *, P < 0.05, vs. normoxia group. Results are shown as mean ± SD

We first assessed the potential effects of hypoxia on MEC by treating isolated MEC with either normoxic or hypoxic conditions for 48 h. Afterward, cells and supernatants were collected for biological assays. Under hypoxic conditions, we found a significant increase for mRNA levels of IL1β, IL6, TNFα, SDF1, VEGF, and HIF1α, while MCP1 expression remained unchanged compared to normoxic conditions (Fig. 2a). Protein levels were then measured by ELISA for IL1β (Fig. 2b), IL6 (Fig. 2c), TNFα (Fig. 2d), MCP1 (Fig. 2e), and SDF1 (Fig. 2f), and by western blotting for VEGF and HIF1α (Fig. 2g, h), revealing a similar pattern to the mRNA expression results. Additionally, hypoxia significantly reduced the GSH/GSSG ratio (Fig. 2i) and potentiated 8-oxo-dG generation (Fig. 2j, k, Fig. S1a), indicating oxidative stress compared to normoxic conditions.

### Hypoxia and MEC-released factors trigger epigenetic modifications, altered gene expression, and redox imbalance in amygdala neurons

**Fig. 3:**
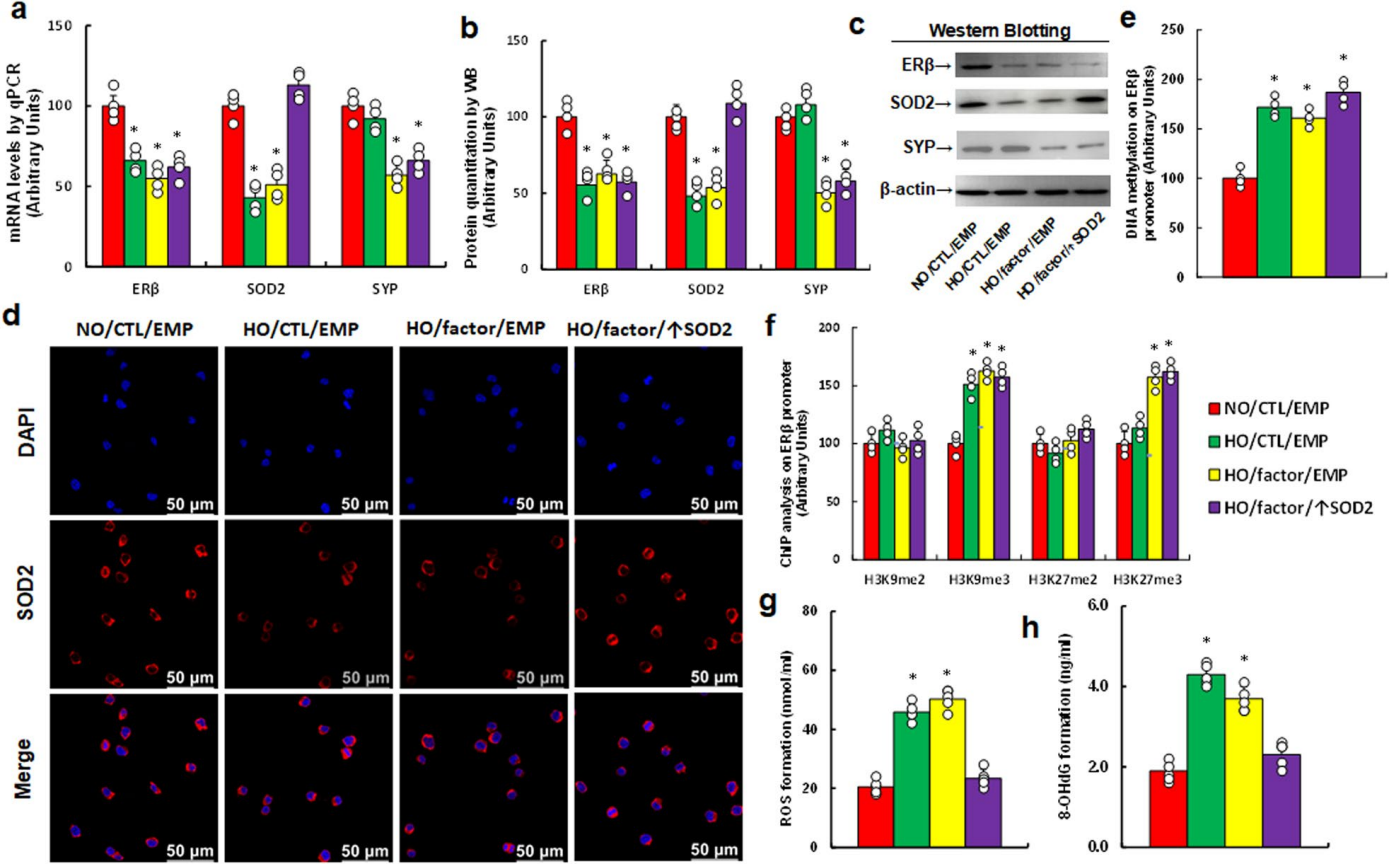
Hypoxia and vascular factors induce epigenetic modifications, altered gene expression, and redox imbalance in amygdala neurons. Amygdala neurons were isolated from untreated mice on E18, then infected with either SOD2 lentivirus (↑SOD2) or an empty control (EMP). Cells were incubated with 50% supernatant from hypoxia-treated (factor) or normoxia-treated (CTL) MEC, under either normoxia (NO) or hypoxia (HO) for 48 h. Treated cells were collected for analysis. **a** mRNA analysis, n = 4; **b** protein quantification, n = 5; **c** representative western blots for **b**; **d** immunostaining images for SOD2; **e** DNA methylation of the ERβ promoter, n = 4; **f** histone methylation on the ERβ promoter, n = 4; **g** ROS formation, n = 5; **h** 8-OHdG formation, n = 5. *, P < 0.05, vs. NO/CTL/EMP group. Results are shown as mean ± SD

To assess the impact of the vascular system on amygdala neurons under hypoxia, we treated the neurons with hypoxia and vascular factors by adding 50% (v/v) supernatant from hypoxia-treated MEC. Hypoxia alone (HO/CTL/EMP) significantly reduced the mRNA levels of ERβ and SOD2, with no effect on SYP. In contrast, hypoxia combined with vascular factors (HO/factor/EMP) significantly reduced the mRNA levels of all three genes compared to the control group (NO/CTL/EMP). SOD2 overexpression (HO/factor/↑SOD2) completely restored SOD2 expression suppressed by hypoxia and vascular factors but had no effect on ERβ or SYP (Fig. 3a). Protein levels followed a similar pattern to the mRNA expression (Fig. 3b, c, Fig. S1b). Immunostaining for SOD2 confirmed these findings (Fig. 3d). We next investigated epigenetic modifications at the ERβ promoter and found that both hypoxia alone (HO/CTL/EMP) and the combination of hypoxia with vascular factors (HO/factor/EMP) significantly increased DNA methylation. SOD2 overexpression (HO/factor/↑SOD2) had no effect on this increase (Fig. 3e). Additionally, both hypoxia alone and the combination of hypoxia with vascular factors significantly increased H3K9me3 and H3K27me3 modifications. Hypoxia alone increased H3K9me3 but showed no effect on H3K27me3 compared to the control (NO/CTL/EMP), and SOD2 overexpression (HO/factor/↑SOD2) did not alter these changes (Fig. 3f). Lastly, we examined redox balance and found that both hypoxia alone and the combination of hypoxia with vascular factors significantly increased ROS (Fig. 3g) and 8-OHdG generation (Fig. 3h) compared with control group. SOD2 overexpression completely reversed these effects. We also assessed H4 methylation (Fig. S2a) and histone acetylation (Fig. S2b), finding no differences among the treatments.

### Hypoxia and vascular factors induce mitochondrial dysfunction in amygdala neurons

**Fig. 4:**
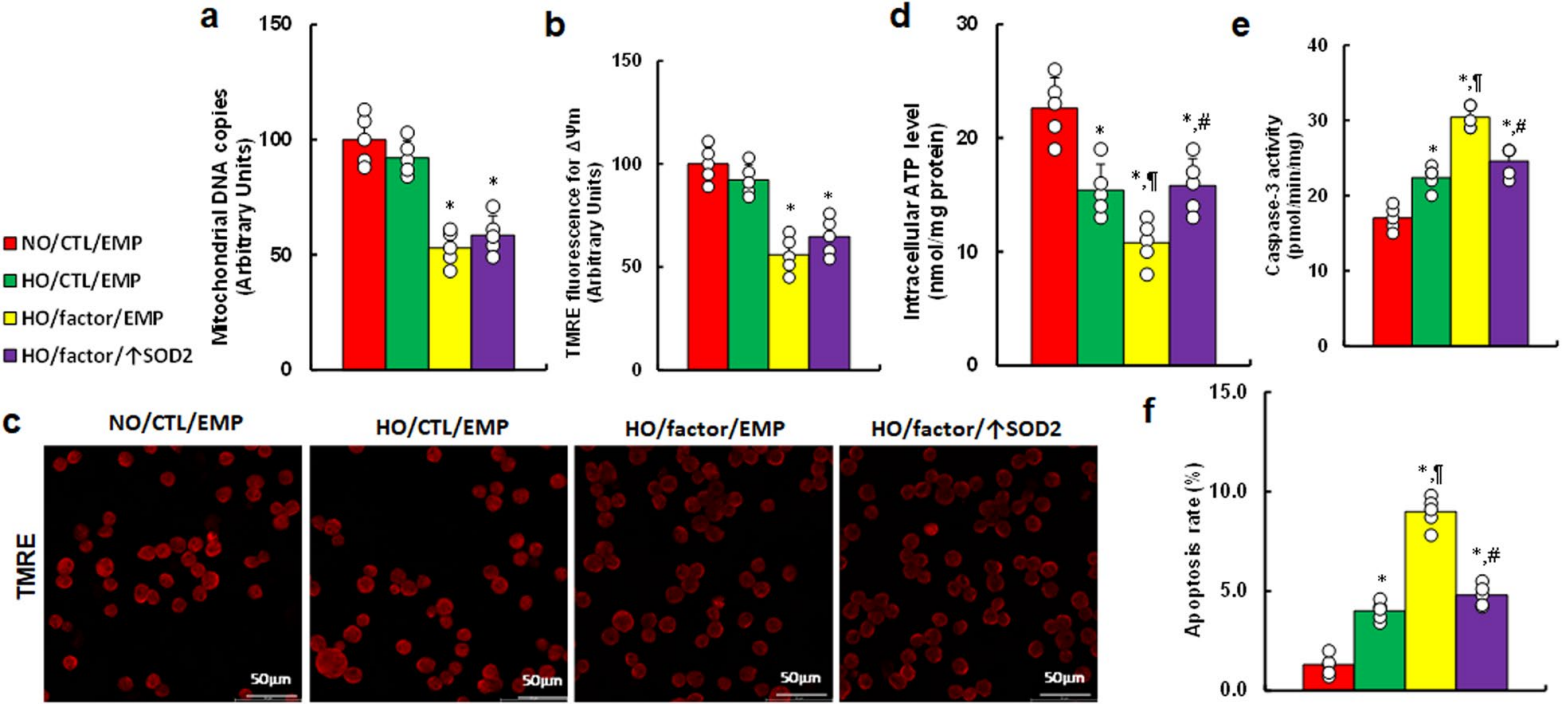
Hypoxia and vascular factors induce mitochondrial dysfunction in amygdala neurons. Amygdala neurons from E18 untreated mice were infected with SOD2 lentivirus (↑SOD2) or an empty control (EMP) for 24 h, then treated with 50% supernatant from hypoxia (factor) or normoxia (CTL)-treated MECs, under normoxia (NO) or hypoxia (HO) for 48 h. Cells were collected for analysis. **a** mitochondrial DNA copies; **b** mitochondrial membrane potential (ΔѰm); **c** representative images for **b**; **d** intracellular ATP levels; **e** caspase-3 activity; **f** apoptosis rate. n = 5. *, P < 0.05, vs. NO/CTL/EMP group; ¶, P < 0.05, vs. HO/CTL/EMP; #, P < 0.05, vs. HO/factor/EMP. Results are shown as mean ± SD

We determined the possible impact of hypoxia and vascular factors on mitochondrial function in these neurons. We found that hypoxia alone (HO/CTL/EMP) showed no effect on mitochondrial DNA copy numbers (Fig. 4a) or mitochondrial membrane potential (ΔѰm) (Fig. 4b, c). However, it decreased intracellular ATP (Fig. 4d) and significantly potentiated caspase-3 activity (Fig. 4e) and apoptosis rate (Fig. 4f) compared with NO/CTL/EMP group. The combination of hypoxia and vascular factors (HO/factor/EMP) further amplified these effects. Notably, increased SOD2 expression (HO/factor/↑SOD2) partially mitigated this enhancement compared to the HO/factor/EMP treatment.

### Perinatal hypoxia induces altered gene expression and oxidative stress in MEC, with ERβ expression in the endothelium partially mitigating these effects in pregnant female mice

**Fig. 5:**
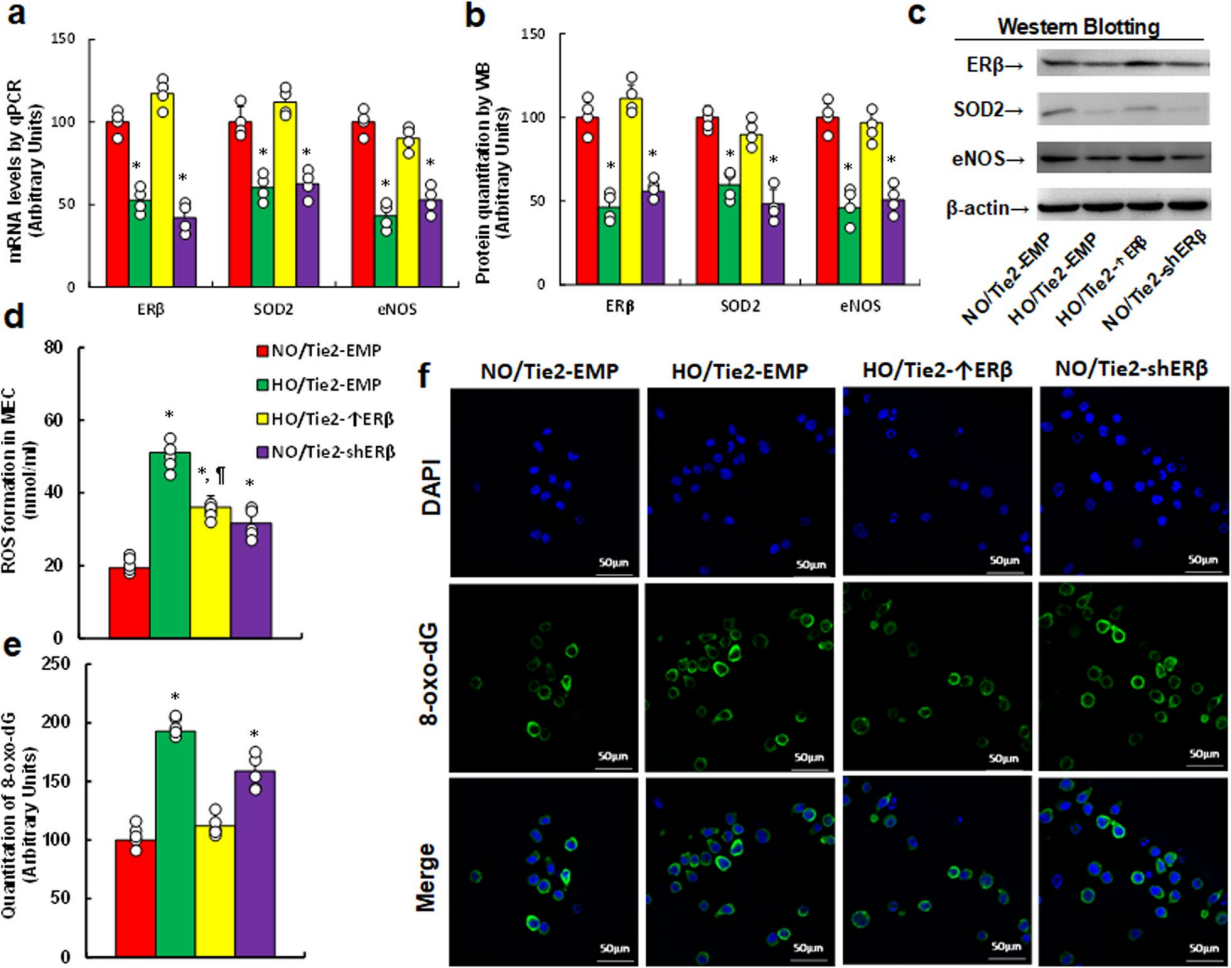
Perinatal hypoxia triggers altered gene expression and oxidative stress in MEC, with ERβ expression in the endothelium partially ameliorating these effects in pregnant mice. Female mice were injected with Tie2-driven ERβ expression lentivirus (Tie2-↑ERβ), ERβ knockdown lentivirus (Tie2-shERβ), or an empty lentivirus control (Tie2-EMP). During pregnancy, mice were exposed to either normoxia (NO) or hypoxia (HO) from E8 to E21. After delivery at P21, dams were euthanized, and MEC cells were isolated for analysis. **a** mRNA analysis, n = 4; **b** protein quantification, n = 5; **c** representative western blots for **b**; **d** ROS formation, n = 7; **e** 8-oxo-dG formation, n = 7; **f** representative images for **e**. *, P < 0.05, vs. NO/Tie2-EMP group; ¶, P < 0.05, vs. HO/Tie2-EMP. Results are shown as mean ± SD

We determined the possible impact of hypoxia on the vascular system in the presence of endothelium-specific ERβ expression, which was modulated by injecting a Tie2-driven ERβ lentivirus. Female mice were injected with the Tie2-driven lentivirus, and following delivery, the pregnant mice were euthanized to isolate MEC for biological analysis. First, we analyzed gene expression from isolated MEC and observed that hypoxia (HO/Tie2-EMP) significantly reduced mRNA levels of SOD2, ERβ, and eNOS compared with normoxia group (NO/Tie2-EMP). However, ERβ overexpression (HO/Tie2-↑ERβ) completely restored these levels, while ERβ knockdown (NO/Tie2-shERβ) mimicked the effects of hypoxia (Fig. 5a). Protein levels were also assessed, revealing a similar expression pattern to the mRNA results (Fig. 5b, c, Fig. S1c). Next, we evaluated redox balance in these cells. Hypoxia (HO/Tie2-EMP) significantly potentiated ROS production (Fig. 5d) and 8-oxo-dG generation (Fig. 5e) compared with normoxia (NO/Tie2-EMP). ERβ overexpression (HO/Tie2-↑ERβ) partially mitigated these effects, while ERβ knockdown (NO/Tie2-shERβ) partially replicated the impact of hypoxia.

### Perinatal hypoxia triggers vascular dysfunction, while ERβ expression in the endothelium partially alleviates this effect in dams at P21

**Fig. 6:**
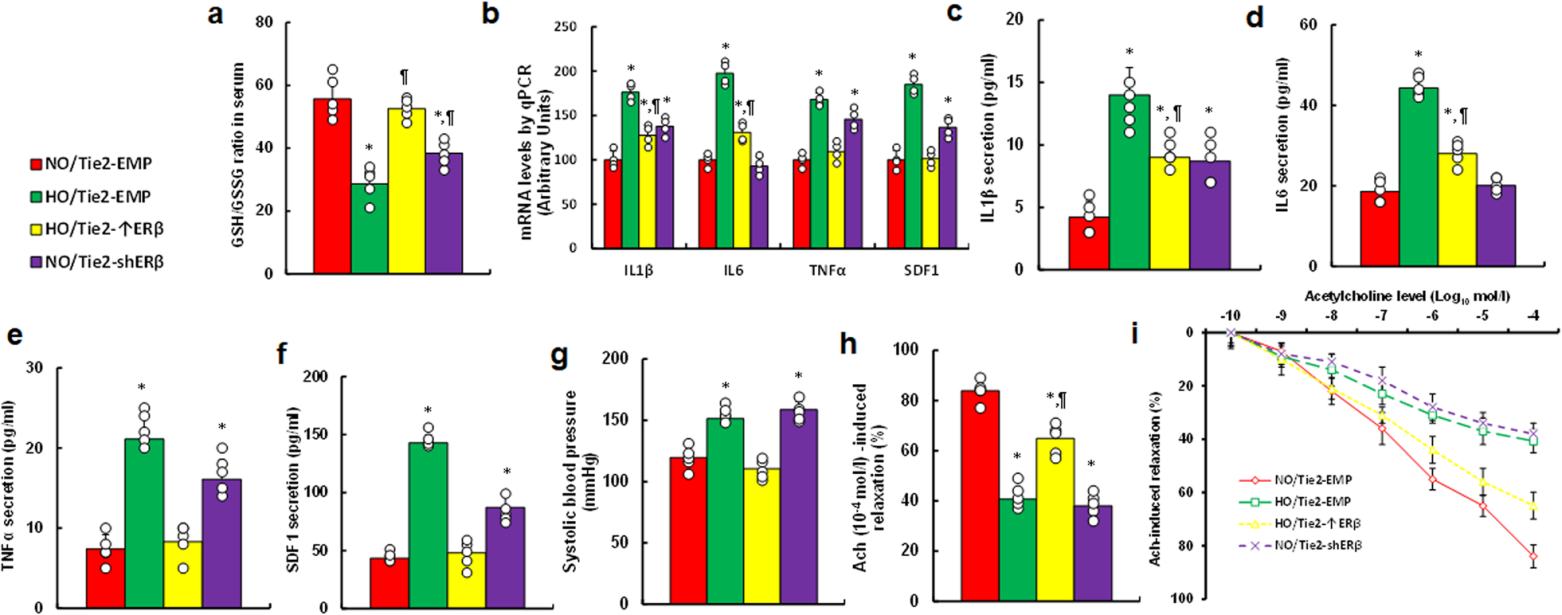
Perinatal hypoxia induces vascular dysfunction, with ERβ expression in the endothelium partially ameliorating these effects in dams at P21. Female mice were injected with Tie2-driven ERβ expression lentivirus (Tie2-↑ERβ), ERβ knockdown lentivirus (Tie2-shERβ), or an empty lentivirus control (Tie2-EMP). During pregnancy, mice were exposed to normoxia (NO) or hypoxia (HO) from E8 to E21. After delivery at P21, biomedical analyses were performed on the dams. **a** GSH/GSSG ratio in serum, n = 7; **b** mRNA analysis in PBMC, n = 4; **c**–**f** serum pro-inflammatory cytokine levels, n = 7: IL1β (**c**), IL6 (**d**), TNFα (**e**), and SDF1 (**f**); **g** systolic blood pressure, n = 7; **h** Ach (10^–4^ mol/l)-induced relaxation, n = 7; **i** Ach-induced dose–response curves, n = 7. *, P < 0.05, vs. NO/Tie2-EMP group; ¶, P < 0.05, vs. HO/Tie2-EMP. Results are shown as mean ± SD

We determined the effects of hypoxia on the circulatory system and vascular function in dams at P21. Hypoxia treatment (HO/Tie2-EMP) significantly decreased the GSH/GSSG ratio in serum compared with normoxia (NO/Tie2-EMP) group. ERβ overexpression (HO/Tie2-↑ERβ) fully restored this ratio, whereas ERβ knockdown (NO/Tie2-shERβ) partially mimicked the hypoxic effect (Fig. 6a). We also measured pro-inflammatory cytokine expression in PBMC. Hypoxia (HO/Tie2-EMP) significantly potentiated mRNA levels of TNFα, IL6, IL1β, and SDF1 compared to the normoxia group. ERβ overexpression (HO/Tie2-↑ERβ) partially reduced these levels, while ERβ knockdown (NO/Tie2-shERβ) partially mimicked the effect of hypoxia (Fig. 6b). Similarly, cytokine levels in serum, including IL1β (Fig. 6c), IL6 (Fig. 6d), TNFα (Fig. 6e), and SDF1 (Fig. 6f), followed the same expression pattern as the mRNA levels. We also measured systolic blood pressure, which was significantly elevated in the hypoxia group (HO/Tie2-EMP) compared to normoxia (NO/Tie2-EMP). ERβ overexpression (HO/Tie2-↑ERβ) reduced blood pressure, while ERβ knockdown (NO/Tie2-shERβ) replicated the hypoxic effect (Fig. 6g). Additionally, hypoxia (HO/Tie2-EMP) impaired aortic relaxation in response to Ach, compared to normoxia (NO/Tie2-EMP). ERβ overexpression (HO/Tie2-↑ERβ) partially improved relaxation, while ERβ knockdown (NO/Tie2-shERβ) replicated the impairment (Fig. 6h, i).

### Exposure to perinatal hypoxia induces oxidative stress and triggers the release of pro-inflammatory cytokines in the vascular system of offspring, with endothelial ERβ expression partially mitigating these effects

**Fig. 7:**
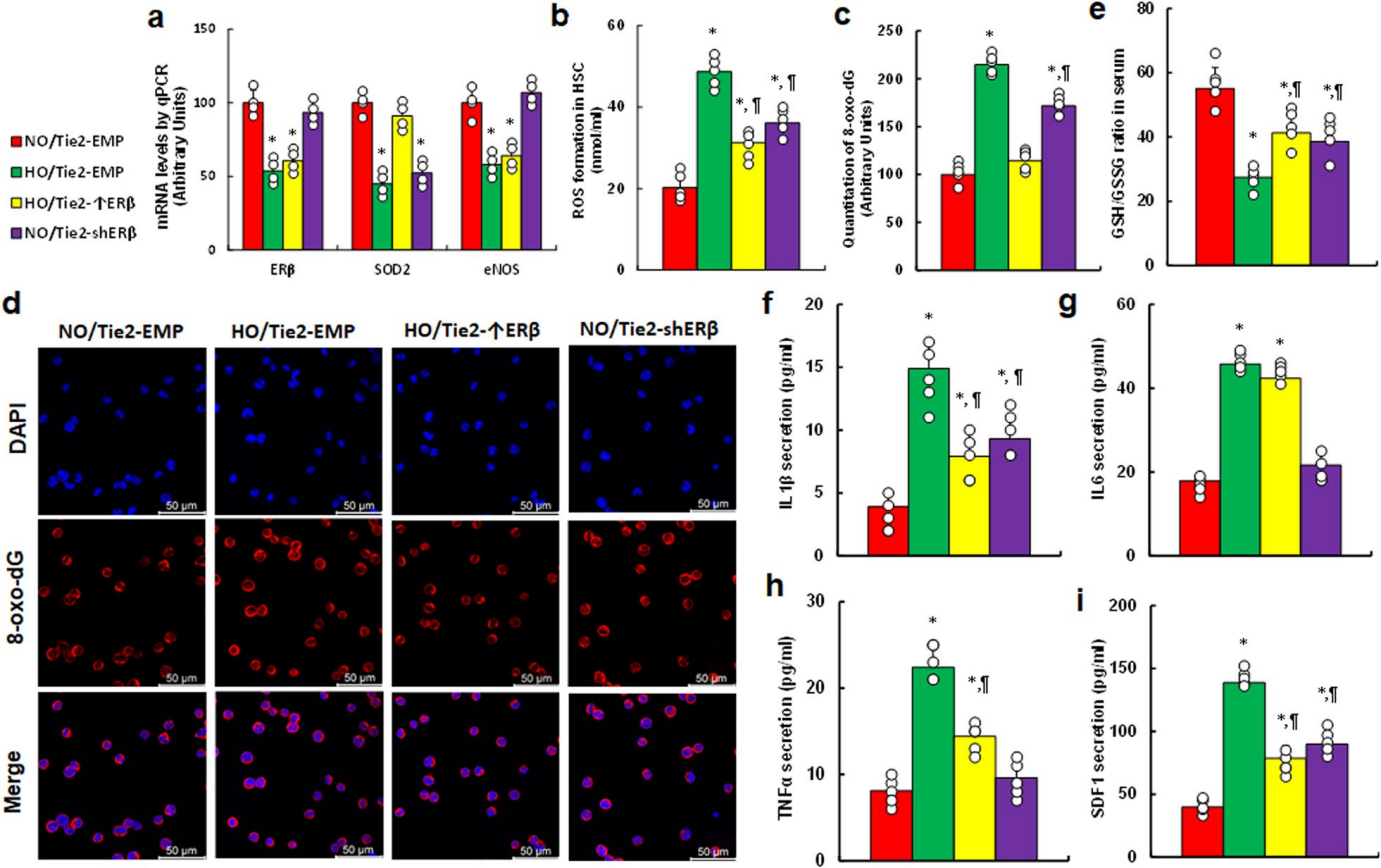
Perinatal hypoxia exposure induces oxidative stress and the release of pro-inflammatory cytokines in the vascular system of offspring, with endothelial ERβ expression partially mitigating these effects. Female mice were injected with lentiviruses encoding either endothelial-specific ERβ overexpression (Tie2-↑ERβ), ERβ knockdown (Tie2-shERβ), or an empty control vector (Tie2-EMP). Mice were subjected to normoxia (NO) or hypoxia (HO) from embryonic day 8 (E8) to E21. Offspring were maintained in normoxia after postnatal day 8 (P8) and analyzed at P30. **a**–**d** Hematopoietic stem cells (HSC) were isolated for biochemical assays: **a** mRNA levels, n = 4. **b** ROS production, n = 7. **c** 8-oxo-dG formation, and **d** representative images of **c**, n = 7. **e** serum GSH/GSSG ratio, n = 7. **f**–**i** serum levels of pro-inflammatory cytokines, n = 7: IL1β (**f**), IL6 (**g**), TNFα (**h**), and SDF1 (**i**). *, P < 0.05 vs. NO/Tie2-EMP group; ¶,P < 0.05 vs. HO/Tie2-EMP. Data are presented as mean ± SD

**Fig. 8:**
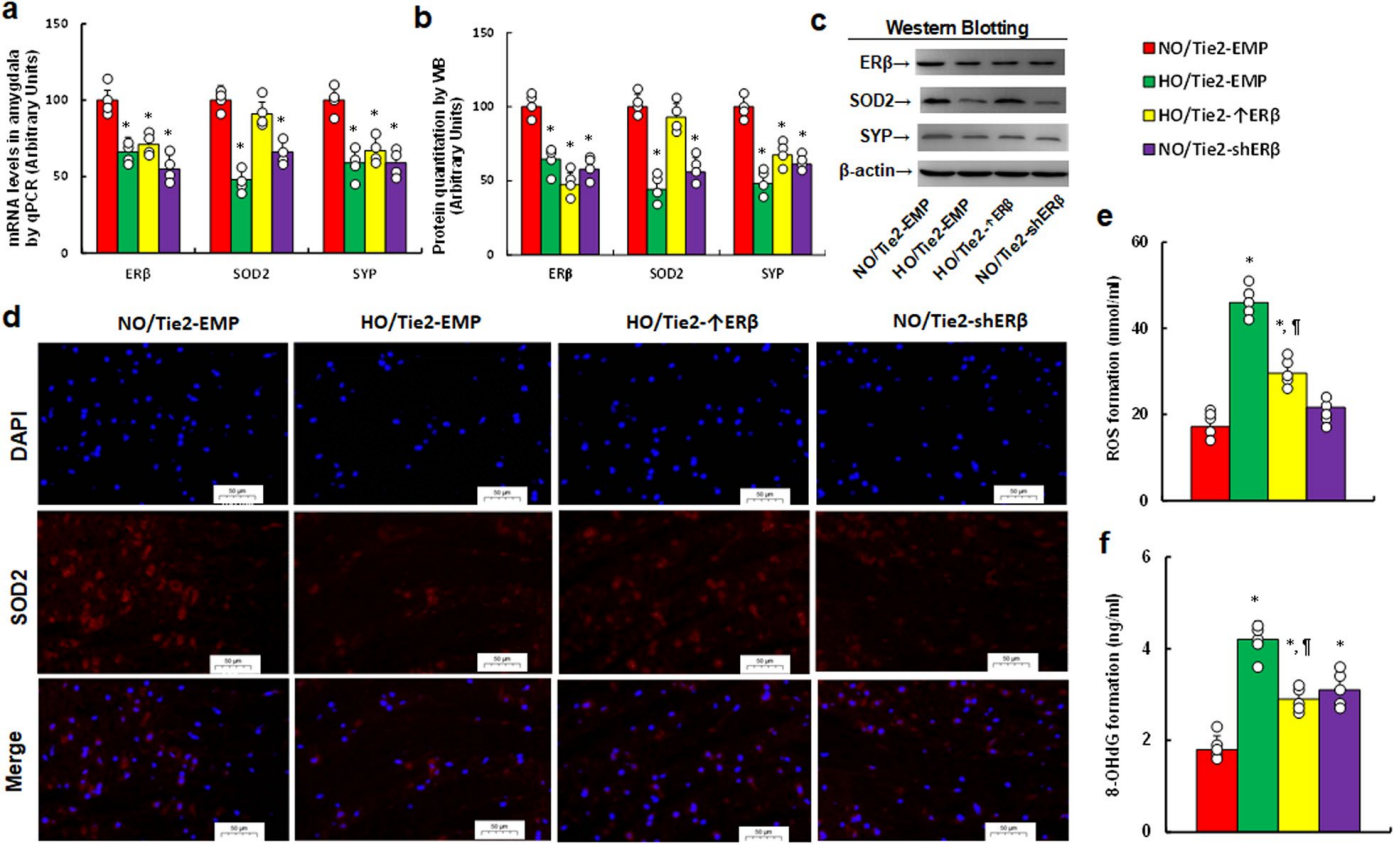
Perinatal hypoxia exposure alters gene expression and oxidative stress in offspring brain tissues, with ERβ expression in endothelium partially ameliorating these effects. Female mice were injected with Tie2-driven ERβ expression lentivirus (Tie2-↑ERβ), ERβ knockdown lentivirus (Tie2-shERβ), or an empty lentivirus control (Tie2-EMP). Mice were exposed to normoxia (NO) or hypoxia (HO) from E8 to E21. Offspring were raised in normoxia after P8 and used for further analysis at P30. **a** mRNA levels in the amygdala, n = 4; **b** protein quantification in the amygdala, n = 5; **c** representative western blots for **b**; **d** immunohistochemistry images for SOD2 in amygdala tissues; **e** ROS formation, n = 7; **f** 8-OHdG formation, n = 7. *, P < 0.05, vs. NO/Tie2-EMP group; ¶, P < 0.05, vs. HO/Tie2-EMP. Results are shown as mean ± SD

We first examined the impact of hypoxia on HSC, where perinatal hypoxia significantly reduced the mRNA levels of ERβ, SOD2, and eNOS. ERβ overexpression (HO/Tie2-↑ERβ) fully restored these levels, while ERβ knockdown (NO/Tie2-shERβ) mimicked the hypoxic reduction of SOD2 without affecting ERβ or eNOS expression (Fig. 7a). We also assessed redox balance in HSC, finding that perinatal hypoxia significantly increased ROS production (Fig. 7b) and 8-oxo-dG levels (Fig. 7c, d); ERβ overexpression partially reversed these effects, whereas ERβ knockdown partially mimicked the hypoxic impact. Additionally, hypoxia exposure in offspring serum was linked to a significantly lower GSH/GSSG ratio (Fig. 7e) and elevated cytokine release, including IL1β (Fig. 7f), IL6 (Fig. 7g), TNFα (Fig. 7h), and SDF1 (Fig. 7i). ERβ overexpression partially reversed these changes, while ERβ knockdown partially replicated the hypoxic effects.

### Perinatal hypoxia alters gene expression and redox balance in offspring brain tissues, with ERβ expression in the endothelium partly mitigating these effects

We evaluated the effects of perinatal hypoxia and ERβ expression on brain tissues in offspring. In the amygdala, hypoxia (HO/Tie2-EMP) significantly reduced mRNA levels of SOD2, ERβ, and SYP compared with normoxia (NO/Tie2-EMP). ERβ overexpression (HO/Tie2-↑ERβ) fully restored SOD2 expression but had no effect on ERβ and SYP. ERβ knockdown (NO/Tie2-shERβ) completely mimicked the hypoxic effects (Fig. 8a). Protein levels showed a similar pattern (Fig. 8b, c, Fig. S1d). Immunohistochemistry (IHC) also confirmed that SOD2 protein levels mirrored the mRNA data (Fig. 8d). We further examined redox balance in the amygdala, where hypoxia (HO/Tie2-EMP) significantly increased ROS (Fig. 8e) and 8-OHdG formation (Fig. 8f) compared to normoxia. ERβ overexpression (HO/Tie2-↑ERβ) partially alleviated oxidative stress, whereas ERβ knockdown (NO/Tie2-shERβ) partially replicated the hypoxic effect on 8-OHdG formation but had no impact on ROS production. In the hypothalamus, hypoxia (HO/Tie2-EMP) significantly reduced SOD2 and SYP mRNA levels compared to normoxia. ERβ overexpression restored SOD2 levels but had no effect on SYP, and ERβ knockdown had no effect on either gene (Fig. S3a). In the hippocampus, hypoxia significantly reduced SOD2 expression, which was fully restored by ERβ overexpression. Neither ERβ knockdown nor any treatment affected mRNA levels of ERβ or SYP (Fig. S3b).

### Perinatal hypoxia alters behavioral outcomes in offspring, with ERβ expression in the endothelium partially mitigating these effects

**Fig. 9:**
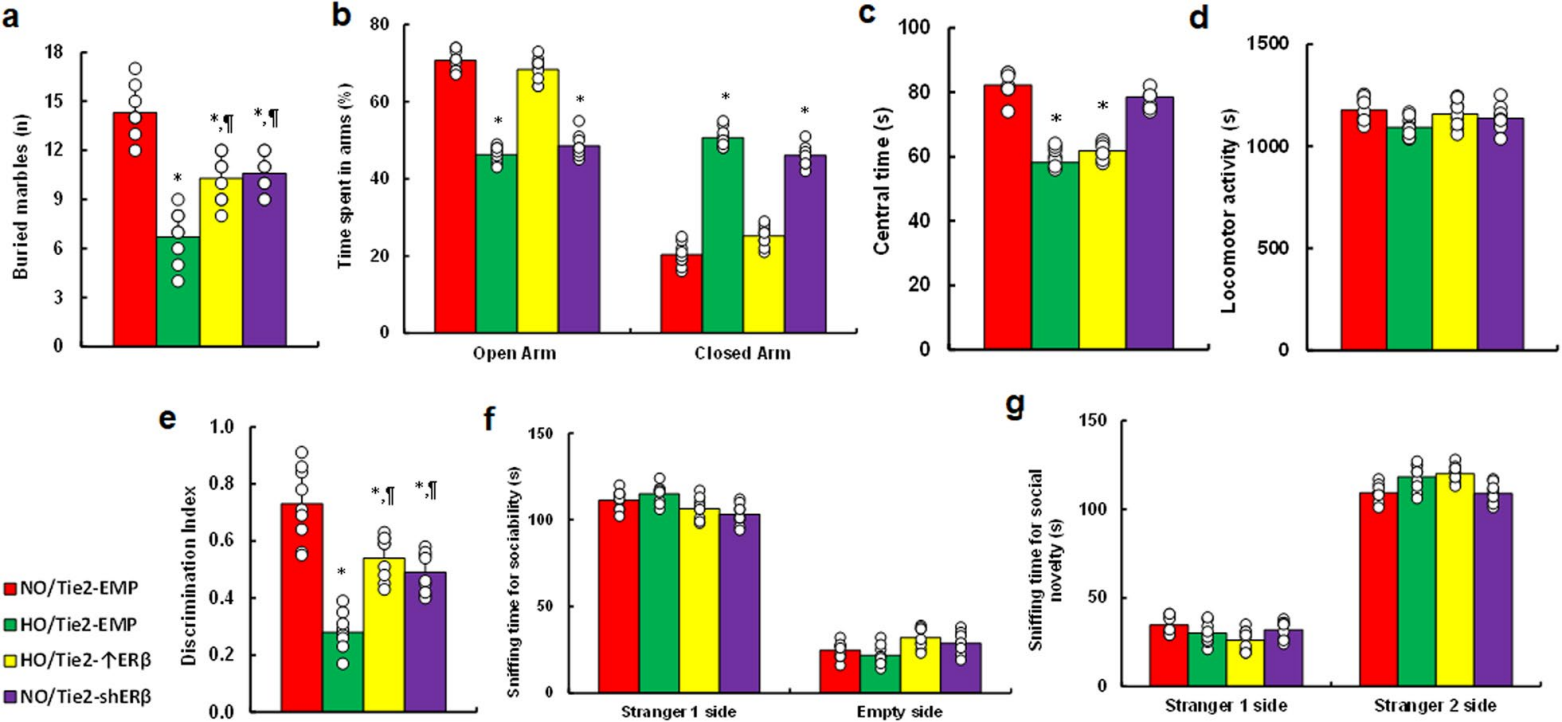
Perinatal hypoxia exposure induces altered animal behaviors in offspring, with ERβ expression in endothelium partially ameliorating these effects. Female mice were injected with Tie2-driven ERβ expression lentivirus (Tie2-↑ERβ), ERβ knockdown lentivirus (Tie2-shERβ), or an empty lentivirus control (Tie2-EMP). During pregnancy, mice were exposed to normoxia (NO) or hypoxia (HO) from E8 to E21. Offspring were raised in normoxia after P8 and underwent behavioral tests at P30. **a** marble-burying test; **b** elevated plus maze (EPM) test; (c,d) open-field test: time spent in the central area (**c**) and total locomotor activity (**d**); **e** discrimination index in the novel object recognition (NOR) test; **f**, **g** three-chambered social test for sociability (**f**) and social novelty (**g**). n = 9. *, P < 0.05, vs. NO/Tie2-EMP group; ¶, P < 0.05, vs. HO/Tie2-EMP. Results are shown as mean ± SD

In Marble-burying test, hypoxia (HO/Tie2-EMP) significantly reduced the number of buried marbles compared to normoxia. ERβ overexpression (HO/Tie2-↑ERβ) partially restored this behavior, while ERβ knockdown (NO/Tie2-shERβ) partially mimicked the hypoxic effect (Fig. 9a). In Elevated Plus Maze (EPM), hypoxia-exposed mice (HO/Tie2-EMP) spent less time in Open Arm and more time in Closed Arm compared to normoxia. ERβ overexpression restored this behavior, while ERβ knockdown mimicked the hypoxic response (Fig. 9b). In Open-field test, hypoxia-exposed mice spent shorter time in the center area compared to normoxia, with no effect observed from ERβ overexpression or knockdown on this behavior (Fig. 9c). Total locomotor activity was unaffected by all treatments (Fig. 9d). In the novel object recognition (NOR) test, hypoxia (HO/Tie2-EMP) reduced the discrimination index compared to normoxia. ERβ overexpression partially restored this, while ERβ knockdown partially mimicked the hypoxic effect (Fig. 9e). Finally, in the three-chamber social test, no differences in sociability (Fig. 9f) or social novelty (Fig. 9g) were observed across treatments.

## Discussion

In this study, we found that hypoxia in MEC led to increased release of pro-inflammatory factors, including IL1β, IL6, TNFα, SDF1, and VEGF. When these released factors were introduced to isolated amygdala neurons under hypoxic conditions, the neurons exhibited decreased gene expression of SOD2, ERβ, and SYP, along with disrupted redox balance, impaired mitochondrial function, and epigenetic modifications on the ERβ promoter. ERβ expression partially ameliorated these effects, while ERβ knockdown mimicked the impact of hypoxia. In a congenital heart disease (CHD) mouse model, hypoxia exposure triggered vascular dysfunction in pregnant dams, altered gene expression and redox balance in brain tissues, and affected behaviors in offspring. Prenatal ERβ expression in the endothelium partially mitigated the effect, while ERβ knockdown partly replicated hypoxia-induced damage.

### Effects of prenatal ERβ expression in endothelium

ERβ is known to regulate redox balance and mitochondrial function through SOD2 and ERRα (Li et al. 2015; Liu et al. 2014). In our study, ERβ expression in amygdala neurons reduced hypoxia-induced oxidative stress, mitochondrial dysfunction, and epigenetic changes in vitro. In the CHD mouse model, Tie2-driven ERβ lentivirus injections were used to modulate endothelial ERβ expression (Kong et al. 2016; Zhan et al. 2016). Results demonstrated that ERβ expression in the endothelium ameliorated hypoxia-induced vascular dysfunction, including oxidative stress, elevated blood pressure (Svitok et al. 2016), and reduced aortic relaxation (Zhan et al. 2016) in pregnant dams. Conversely, ERβ knockdown in the endothelium mimicked the hypoxic effects. Interestingly, endothelial ERβ expression during pregnancy had a protective effect against neurodevelopmental abnormalities in offspring exposed to perinatal hypoxia (E16–P8). ERβ-mediated regulation of oxidative stress and cytokine release likely triggers epigenetic changes (Chen et al. 2019) in neurons, contributing to neurodevelopmental protection even under hypoxic conditions.

### Hypoxia-mediated vascular damage during pregnancy

In vitro studies demonstrated that hypoxia exposure induces pro-inflammatory cytokines release of Bartels et al. (2013), Price et al. (2024), activates HIF1α signaling, and triggers oxidative stress in MEC (Ceradini et al. 2008). In vivo, hypoxia suppressed the expression of ERβ, SOD2, and eNOS, leading to redox imbalances, increased blood pressure, and impaired aortic relaxation (Kong et al. 2016; Zhan et al. 2016). Endothelial ERβ expression partially restored redox balance, cytokine release (including IL1β, IL6, TNFα, SDF1, and VEGF), and protected against vascular damage. This restoration of cytokine balance may prevent epigenetic alterations in fetal development, explaining the protective effect of endothelial ERβ expression against perinatal hypoxia-mediated neurodevelopmental abnormalities.

### Hypoxia-induced vascular dysfunction in offspring

Our in vivo study revealed that perinatal hypoxia from E16 to P8 suppresses gene expression, including ERβ, SOD2, and eNOS, and induces oxidative stress in HSC. Previously, we found that oxidative stress-induced epigenetic modifications and gene suppression in HSC can be inherited by PBMC during subsequent differentiation (Zeng et al. 2022; Lu et al. 2020). This explains the observed reduction in the GSH/GSSG ratio and the increased release of cytokines such as IL1β, IL6, TNFα, and SDF1 in peripheral blood. Hypoxia is known to disrupt fetal and neonatal vascular development by impairing angiogenic signaling pathways, reducing endothelial cell proliferation, and altering vascular remodeling (Krock et al. 2011; Humar et al. 2002). Studies indicate that hypoxia-induced dysregulation of VEGF and Notch signaling affects vessel integrity and function, leading to long-term vascular abnormalities (Holderfield and Hughes 2008). Additionally, the release of these vascular factors may exacerbate neurodevelopmental abnormalities by crossing the blood–brain barrier (Gong and Jia 2022; He et al. 2020).

### Perinatal hypoxia and neurodevelopmental abnormalities

In vitro studies on amygdala neurons, chosen for their critical role in neurodevelopment (Wang et al. 2019; Zou et al. 2017), mimicked hypoxic conditions and added MEC-derived cytokines. These factors, which can pass through the placenta, triggered epigenetic changes (Coppin et al. 2023; Czamara et al. 2021), altered gene expression, disrupted redox balance, and mitochondrial dysfunction in neurons. In vivo, a perinatal hypoxia mouse model (E16–P8) was used to simulate the timeline of cyanotic CHD from late gestation to postnatal development. Perinatal hypoxia altered the expression of ERβ, SOD2, and SYP, impaired redox balance, and led to abnormal behaviors in offspring. Prenatal endothelial ERβ expression partially ameliorated these effects, indicating that preventive interventions during pregnancy may mitigate neurodevelopmental damage caused by perinatal hypoxia (Romanowicz et al. 2019; Orzel et al. 2023).

### Limitations

The study relies on a CHD mouse model, which may not fully replicate the complexity and heterogeneity of cyanotic congenital heart disease in human populations. Translational relevance to humans should be interpreted cautiously. Also, the study primarily examines the amygdala neurons, potentially overlooking the effects of hypoxia on other brain regions critical for neurodevelopment, such as the hippocampus or cortex. In addition, endothelial cell-derived factors are implicated, the study does not comprehensively assess other vascular mechanisms, such as blood–brain barrier integrity, that might contribute to neurodevelopmental abnormalities. Furthermore, the study does not address potential sex-based differences in response to hypoxia or ERβ modulation, which could influence neurodevelopmental outcomes. By addressing these limitations in future studies, the findings can be better contextualized and potentially translated to improve the understanding and management of neurodevelopmental abnormalities in children with CHD.

This study primarily evaluated endothelial cell function and vascular dysfunction at P21, which may not fully capture the critical developmental events occurring during the fetal (E16–E18) and neonatal (P1–P8) stages. Given that vascular formation is most active between E9.5 and E18, assessing these earlier stages could provide more direct insights into the timing and mechanisms of ERβ’s effects on endothelial cells. However, prior research has demonstrated that hypoxia disrupts key angiogenic pathways, including VEGF and Notch signaling, leading to long-term vascular abnormalities (Tamblyn et al. 2013). Hypoxia-inducible factors regulate VEGF expression, influencing angiogenesis and vascular permeability, which further contributes to endothelial dysfunction (O’Keeffe and Kenny 2014). Our findings at P21 suggest that these impairments likely originate during fetal and early postnatal development, consistent with existing literature on prenatal hypoxia-induced epigenetic modifications and endothelial dysfunction (Win et al. 2018).

Similarly, while we focused on vascular and neurodevelopmental changes in the amygdala at P21, additional analyses at earlier developmental stages could further elucidate the causal relationship between hypoxia-induced vascular dysfunction and neuronal alterations. Hypoxia-induced inflammatory changes, such as increased cytokine levels observed in our study, likely play a role in disrupting normal amygdala development (McAdams and Juul 2012). The interplay between VEGF, Notch, and TGFβ signaling pathways in vascular development and remodeling further underscores the complexity of these hypoxia-induced alterations (Holderfield and Hughes 2008). While these findings support the link between vascular dysfunction and neurodevelopmental abnormalities, future studies are needed to examine these effects across multiple time points for a more comprehensive understanding.

### Future directions

Future research should explore the role of vascular-endothelial-derived factors, such as VEGF and SDF1, in fetal brain development. VEGF has been shown to regulate neuronal migration and blood vessel maturation, both essential for proper cortical formation. Furthermore, the interaction between VEGF and SDF1 enhances endothelial progenitor cell migration and proliferation, suggesting a coordinated role in neurovascular development (Tamblyn et al. 2013; O’Keeffe and Kenny 2014). Investigating these mechanisms could provide critical insights into how vascular abnormalities contribute to neurodevelopmental disorders, paving the way for potential therapeutic interventions.

## Conclusions

Hypoxia, in conjunction with cytokines released from the vascular system, triggers altered gene expression, redox imbalance, mitochondrial dysfunction, and epigenetic modifications in neurons. Perinatal hypoxia from E16 to P8 induces persistent vascular damage in pregnant dams, which remains evident at P21. In offspring, it leads to vascular dysfunction, gene suppression, oxidative stress in brain tissues, and behavioral abnormalities. Notably, prenatal endothelial ERβ expression partially mitigated these effects, highlighting the potential importance of preventive interventions during pregnancy in reducing perinatal hypoxia-induced neurodevelopmental abnormalities in CHD.

## Supplementary Information

Supplementary Material 1.

## Data Availability

No datasets were generated or analysed during the current study.

## Abbreviations

Ach: Acetylcholine
ChIP: Chromatin immunoprecipitation
eNOS: Endothelial nitric oxide synthase
ERβ: Estrogen receptor β
HIF1α: Hypoxia-inducible factor 1α
HO: Hypoxia
HSC: Hematopoietic stem cells
IHC: Immunohistochemistry
IL6: Interleukin-6
IL1β: Interleukin-1β
MCP1: Monocyte chemoattractant protein-1
MEC: Mouse aorta endothelial cells
NO: Normoxia
ROS: Reactive oxygen species
SDF1: Stromal cell-derived factor 1
SOD2: Superoxide dismutase 2
SYP: Synaptophysin
TNFα: Tumor necrosis factor α
VEGF: Vascular endothelial growth factor
ΔѰm: Mitochondrial membrane potential
