# Supplementary material for: Perinatal hypoxia-mediated neurodevelopment abnormalities in congenital heart disease mouse model
Source: Mol Med. 2025 Mar 21;31:109. doi: 10.1186/s10020-025-01158-w (PMC11927194; doi:10.1186/s10020-025-01158-w)
Supplement: Supplementary file 1 — Supplementary Material 1. [file 10020_2025_1158_MOESM1_ESM.docx]

**Perinatal Hypoxia-Mediated Neurodevelopment Abnormalities in Congenital Heart Disease Mouse Model**

**Supplemental Information**

Data S1. MATERIALS AND METHODS

**Reagents and materials**. The antibodies for β-actin (sc-47778, 1:500), eNOS (sc-654, 1:200), ERβ (sc-137381, 1:100), HIF1α (sc-10790, 1:200), SDF1 (sc-28876, 1:500), SOD2 (sc-30080, 1:50 for IHC, 1:200 for WB), SYP (sc-17750, 1:200) and VEGF (sc-507, 1:200) were obtained from Santa Cruz Biotechnology. Antibodies for acetyl-histone H4 K5, K8, K12, and K16 (H4K5,8,12,16ac, #PA5-40084, 1:500) were obtained from Invitrogen. Antibodies for anti-histone H3 acetyl K9, K14, K18, K23, K27(H3K9,14,18,23,27ac, ab47915, 1:500), H4K20me1 (ab9051, 1:500), H4K20me3 (ab9053, 1:500), H4R3me1 (ab17339, 1:500), H3K9me2 (ab1220, 1:500), H3K9me3 (ab8898, 1:500), H3K27me2 (ab24684, 1:500) and H3K27me3 (ab6002, 1:500) were obtained from Abcam. Protein concentration was measured using the Coomassie Protein Assay Kit (Pierce Biotechnology). Hypoxia was induced for 48 hours prior to analysis in a hypoxic chamber in which O2 was removed by flushing with 95% CO2 plus 5% N2 gas for 15 min.

**Isolation of mouse aorta endothelial cells (MEC).** Isolation of MEC from aortas was performed as previously described with minor modifications (1). Briefly, the aorta was isolated and rinsed 3 times with PBS solution, and then were placed in a 100-mm culture dish filled with ice-cold serum-free DMEM medium. The aorta was cut opened, rinsed with serum-free DMEM and placed intimal side down on a sterile plate containing 0.2% collagenase type I. Incubation was performed at 37°C for 30 min. MEC were gathered using a cell scraper and rinsed with 5ml of DMEM plus serum to arrest the digestion process. The medium containing detached MEC was collected and centrifuged at 2000rpm for 10 min. The cell pellet was washed twice, suspended in DMEM medium plus antibiotics and endothelial supplements and placed in a 60-mm culture dish. The medium was changed every other day. The cells were subcultured as long as a monolayer was formed. The isolated endothelial cells were characterized by immunofluorescence staining using an antibody to the von Willebrandt factor (vWF, sc-59810 from Santa Cruz Biotechnology), and the passage P3-P5 MEC was used in this study (2).

**In vitro primary culture of amygdala neurons**. Amygdala tissues were dissected from untreated mouse on embryonic day 18 (E18). Tissues were treated with 0.05% trypsin EDTA for 15 min at 37°C. Trypsin EDTA was replaced with soybean trypsin inhibitor (Sigma) for 5 min at 37°C to stop the reaction. This was then replaced with supplemented Neurobasal A (Invitrogen) followed by mechanical dissociation. Cells were then resuspended in culture media, including Neurobasal A, B27, 1×GlutaMAX and 100 U/ml Pen/Strep (from Invitrogen), and then the cells were incubated at 37°C, 5% CO2 (10). The isolated amygdala neurons were used for further biological assays (3).

**Isolation of mouse PBMC cells**. Heparinized peripheral blood was collected from mouse subjects by heart puncture and diluted 1:3 with Hank's balanced salts solution without Ca^2+^/Mg^2+^ (HBSS solution). The diluted blood was layered onto 10 ml of Ficoll-Paque in 15 ml sterile centrifuge tubes followed by centrifugation at 300×g at 20ºC for 40 min. The PBMC layers were then harvested and washed by HBSS solution. The pellets were resuspended with lysing buffer containing 150 mM NH4Cl, 1.0 mM KHCO3, and 0.1 mM Na2EDTA, pH 7.4 and incubated for 5 min at room temperature to remove contaminated red cells. The cell suspensions were then centrifuged and washed with HBSS solution before the cell pellet was resuspended for further biomedical analysis.

**RT reaction and real-time quantitative PCR.** Total RNA from treated cells was extracted using the RNeasy Micro Kit (Qiagen), and the RNA was reverse transcribed using an Omniscript RT kit (Qiagen). All the primers were designed using Primer 3 Plus software with the Tm at 60°C, primer size of 21 bp, and product length in the range of 140-160 bp (see Table S1). The primers were validated with an amplification efficiency in the range of 1.9-2.1 and the amplified products were confirmed with agarose gel. Real-time quantitative PCR was run on iCycler iQ (Bio-Rad) using the Quantitect SYBR green PCR kit (Qiagen). The PCR was performed by denaturing at 95°C for 8 min followed by 45 cycles of denaturation at 95°C, annealing at 60°C, and extension at 72°C for 10 s, respectively. 1 µl of each cDNA was used to measure target genes. β-actin was used as the housekeeping gene for transcript normalization, and the mean values were used to calculate relative transcript levels with the ^ΔΔ^CT method per instructions from Qiagen. In brief, the amplified transcripts were quantified by the comparative threshold cycle method using β-actin as a normalizer. Fold changes in gene mRNA expression were calculated as 2^−ΔΔCT^ with CT = threshold cycle, ΔCT=CT (target gene)-CT(β-actin), and the ΔΔCT =ΔCT (experimental)-ΔCT (reference).

**Chromatin immunoprecipitation (ChIP).** Cells were washed and crosslinked using 1% formaldehyde for 20 min and terminated by 0.1M glycine. Cell lysates were sonicated and centrifuged. 500 µg of protein were pre-cleared by BSA/salmon sperm DNA with preimmune IgG and a slurry of Protein A Agarose beads. Immunoprecipitations were performed with the indicated antibodies, BSA/salmon sperm DNA and a 50% slurry of Protein A agarose beads. Input and immunoprecipitates were washed and eluted before then being incubated with 0.2 mg/ml Proteinase K for 2 h at 42˚C, followed by 6 h at 65˚C to reverse the formaldehyde crosslinking. DNA fragments were recovered through phenol/chloroform extraction and ethanol precipitation. A ~150 bp fragment on the related promoter was amplified by real-time PCR (qPCR) using the primers provided in Table S1.

**Western blotting.** Cells were lysed in an ice-cold lysis buffer (0.137M NaCl, 2mM EDTA, 10% glycerol, 1% NP-40, 20mM Tris base, pH 8.0) with protease inhibitor cocktail (Sigma). The proteins were separated in 10% SDS-PAGE and transferred to the PVDF membrane, which was then blotted using primary antibodies and then simultaneously incubated with the differentially labeled species-specific secondary antibodies (1:10,000), anti-RABBIT IRDye™ 800CW (green) and anti-MOUSE (or goat) ALEXA680 (red). Membranes were scanned and quantitated using the ODYSSEY Infrared Imaging System (LI-COR, NE) (4).

**Immunostaining**. The treated cells were transferred to cover slips coated with 0.1% gelatin, fixed by 3.7% formaldehyde at 37ºC for 15 min, permeabilized by 1% BSA+0.2% Triton X-100 in PBS for 1 hour, and then blotted with 40 μg/ml (dilute 1:50) of related antibody for either 8-oxo-dG or SOD2 for 2 hours. The cells were then washed three times and the FITC/Texas Red labeled anti-mouse/rabbit secondary antibody (1:500) was added for blotting for another 1 hour. After thorough washing, the slides were visualized and photographed; cell nuclei were stained with 4’,6-diamidino-2-phenylindole dihydrochloride (DAPI, #D9542, from Sigma) and the staining was quantitated by Image J.

**DNA methylation analysis**. The DNA methylation on the ERβ promoter was evaluated using a methylation-specific PCR-based method as described previously with minor modifications (5-7). The genomic DNA was extracted from treated cells, and then treated by bisulfite modification through EpiJET Bisulfite Conversion Kit (#K1461, from Fisher). The treated DNA was then amplified using the following primers: methylated primer: forward 5’- aat ttc ggt gtt att att cga aat c-3’ and reverse 5’- aaa ttc taa act taa ccg cgt acg -3’; unmethylated primer: forward 5’- ttt tgg tgt tat tat ttg aaa ttg g -3’ and reverse 5’- aat tct aaa ctt aac cac ata cat c-3’. Product size: 151bp (methylated) & 148bp (unmethylated); CpG island size: 108bp; Tm: 64.1°C (methylated) & 63.3°C (unmethylated). The final DNA methylation results were normalized by DNA unmethylated results as input.

**Measurement of oxidative stress.** Treated cells were seeded in a 96-well plate and incubated with 10μM CM-H2DCFDA (Invitrogen) for 45min at 37°C. Then, the intracellular formation of reactive oxygen species was measured at excitation/emission wavelengths of 485/530nm using a FLx800 microplate fluorescence reader (Bio-Tek), and the data was normalized as arbitrary units (8). The GSH/GSSG ratio was measured using the GSH/GSSG-Glo™ Assay Kit (#V6611, obtained from Promega) per manufacturers’ instructions. 8-OHdG formation was measured using an OxiSelect™ Oxidative DNA Damage ELISA Kit (Cat No. STA320, from Cell Biolabs Inc.) per manufacturers’ instructions and the formation of 8-oxo-dG was determined by immunostaining using primary antibody for 8-oxo-dG (#4354-MC-050, from Novus Biologicals) and quantitated by Image J.

**Measurement of mitochondrial function.**

*Mitochondrial DNA copies.* Genomic DNA was extracted from treated cells using a QIAamp DNA Mini Kit (Qiagen) and the mitochondrial DNA was extracted using the REPLI-g Mitochondrial DNA Kit (Qiagen). The purified DNA was used for the analysis of genomic β-actin (marker of the nuclear gene) and ATP6 (ATP synthase F0 subunit 6, marker of the mitochondrial gene) respectively using the qPCR method as mentioned above. The primers for genomic β-actin are as follows: forward 5’- tgc gtt aca ccc ttt ttc ttg -3’ and reverse: 5’- acc ttc acc gtt cca gtt ttt -3’. Primers for ATP6: forward 5’- cgt aat tac agg ctt ccg aca -3’ and reverse 5’- ctg taa gcc gga ctg cta atg -3’. The mitochondrial DNA copies were obtained from relative ATP6 copies that were normalized by β-actin copies using the ^ΔΔ^CT method (8-10).

*Intracellular ATP level.* The intracellular ATP level was determined using the luciferin/luciferase-induced bioluminescence system. An ATP standard curve was generated at concentrations of 10^-12^-10^-3^M. Intracellular ATP levels were calculated and expressed as nmol/mg protein (8-10).

*Mitochondria membrane potential*. Mitochondrial membrane potential (MMP, Δψm) was measured using TMRE (from Molecular Probes T-669) staining. A 600 μM T-669 stock solution was prepared using DMSO. Cells were grown on coverslips and immersed in 600 nM TMRE for 20 min at 37°C to load them with dye. The labeling medium was then aspirated and the cells were immersed in 150 nM TMRE to maintain the equilibrium distribution of the fluorophore. The coverslips were mounted with live cells onto confocal microscopes to image the cells using 548 nm excitation/573 nm emission filters. The intensity of TMRE fluorescence was measured using Image J software. Data from 10-20 cells were collected for each experimental condition and mean values of fluorescence intensity ± SEM were calculated (10, 11).

*Measurement of apoptosis.* Apoptosis was evaluated by TUNEL assay using the In Situ Cell Death Detection Kit™ (Roche). Cells were fixed in 4% paraformaldehyde and labeled by TUNEL reagents. Stained cells were photographed by a fluorescence microscope and further quantified by FACS analysis (8).

**Analysis of cytokines**. Mouse cytokines, including IL1β, IL6, TNF-α, MCP1 and SDF1 from either serum or in vitro cell culture supernatant, were measured by Mouse IL-1β/IL-1F2 Quantikine ELISA Kit (#MLB00C), Mouse IL-6 Quantikine ELISA Kit (#M6000B), Mouse TNF-alpha Quantikine ELISA Kit (#MTA00B), Mouse CCL2/JE/MCP-1 Quantikine ELISA Kit and Mouse CXCL12/SDF-1 alpha Quantikine ELISA Kit, respectively, according to manufacturers’ instructions from R&D Systems (12, 13).

**Generation of lentivirus**.

*Preparation of SOD2 expression lentivirus.* The cDNA for mouse SOD2 (obtained from Open Biosystems) was subcloned into the pLVX-Puro vector (from Clontech) with the underlined restriction sites using the following primers: SOD2 forward primer: 5’- gtac- ctcgag- atg ttg tgt cgg gcg gcg tgc-3’ (Xho1) and SOD2 reverse primer: 5’- gtac- tctaga- tca ctt ctt gca agc tgt gta -3’ (Xba1). The lentivirus for SOD2 or empty control (CTL) was expressed through Lenti-X™ Lentiviral Expression Systems (from Clontech) per manufacturers’ instructions, and the prepared lentivirus was used for infection of cells for the expression of either empty (EMP) or SOD2 (↑SOD2) (14).

*Tie2-driven ERβ expression lentivirus.* Mouse genomic DNA was purified from treated mice. The endothelium-specific Tie2 promoter (-1.5kb upstream plus the 1^st^ exon) was amplified by PCR and fused with mouse ERβ cDNA (obtained from Open Biosystems). The construct was subcloned into the pLVX-Puro vector (from Clontech) at the restriction sites of Xho1 and Xba1 using the following primers: forward primer: 5’-gtac-ctcgag- ggt ggt tgc agg ctg gct atg-3’ (Xho1); ERβ reverse primer: 5’-gtac-tctaga- tca ctg aga ctg tag gtt ctg -3’ (Xba1). Tie2-empty was expressed through the Lenti-X™ Lentiviral Expression Systems (from Clontech) according to manufacturers’ instructions. The virus was further purified, concentrated and titrated to reach ~2×10^8^ MOI per mL for tail vein injections into experimental mouses.

*Tie2-driven ERβ shRNA lentivirus.* According to our preliminary data from *in vitro* cell culture experiments, the following sequence was confirmed as the most effective to knockdown mouse ERβ: 5’-tgc tgt tga cag tga gcg aag aga gac act gaa gag gaagta gtg aag cca cag atg tac ttc ctc ttc agt gtc tct ctg tgc cta ctg cct cgg a-3’. The related double strand DNA (dsDNA) was synthesized and annealed. They were fused with mouse Tie2 promoter (-1.5kb upstream plus the 1^st^ exon) by BamH1 sticky site at 5’-end and EcoR1 sticky site at 3’-end, and then inserted into the pLVX-shRNA1 vector (from Clontech) using BamH1/EcoR1 restriction sites. Tie2-scrambled (CTL) or Tie2-shERβ lentivirus was expressed through the Lenti-X™ shRNA Expression System (from Clontech) according to manufacturers’ instructions.

**In vivo animal experiments.** The animal protocol conformed to US NIH guidelines (Guide for the Care and Use of Laboratory Animals, No. 85-23, revised 1996) and was reviewed and approved by the Institutional Animal Care and Use Committee. Experimental mice with C57BL/6J background were housed 3 per cage on a 12:12-h light-dark cycle and were given rodent chow and water ad libitum on arrival. 2 months old female mice received tail vein injections of 100µl lentivirus (2×10^8^ MOI) for Tie2-Empty (CTL), Tie2-ERβ↑, or Tie2-shERβ twice within a 2-day interval. This injection procedure was administered again after one month.

**Prenatal hypoxia mouse model.** The female mice (3-month old) were caged with male and the confirmed pregnant mice were separated into the following groups: Group 1, NO-Tie2-EMP, normaxia group with the injection of Tie2-driven empty lentivirus; Group 2, HO-Tie2-EMP, hypoxia group with the injection of Tie2-driven empty lentivirus; Group 3, HO-Tie2-↑ERβ, hypoxia group with the injection of Tie2 driven ERβ expression lentivirus; Group 4, NO-Tie2-shERβ, normoxia group with the injection of Tie2-driven ERβ knockdown lentivirus. The pregnant mice were incubated in a hypoxic chamber starting on embryonic day (E16), during the experiment, the oxygen concentration was maintained, monitored, and recorded continuously with sensors placed inside the chamber to achieve a level of 11±0.5% of O2. Nitrogen gas was used to displace oxygen. Dams gave birth in the hypoxic chamber (n=9) and pups remained in hypoxia until postnatal day (P8), strain and age-matched normoxia dams (n=9) were kept in normoxia and gave birth under normoxia conditions. After P8, hypoxic animals were moved to normoxia conditions and allowed to recover until further testing at P30. After the baby delivery on postnatal day 21 (p21) when the weaning process was completed, dams were euthanized via decapitalization, the blood was collected for isolation of serum and PBMC, and the MEC were isolated from the thoracic aorta for in vitro cell culture biological assays, and the carotid arteries were isolated to measure vessel tension, while the offspring were used for further analysis at the end of the study on P30. After the evaluation of animal behaviors as described below, the offspring were euthanized, the serum and hematopoietic stem cells (HSC) were prepared for evaluation of vascular function, and the brain tissues, including amygdala, hypothalamus and hippocampus, were isolated for analysis of gene expression and oxidative stress, approximate equal distribution of males and females were used for subsequent offspring experiments, and multiple litters were used for each end point (see details in Fig 1) (15).

**Animal behavior test.** The animal behavior was evaluated by marble-burying test (MBT) (16-19), elevated plus maze (EPM) test (18, 20), open-field test (OFT) (18), new object recognition (NOR) test (21) and three-chambered social test (3, 22).

Marble-burying test (MBT). The experimental mouse was placed in a clean cage (35×23×19 cm^3^) that was filled with wood chip bedding (5 cm depth) containing 20 of colored 1cm-diameter glass marbles with a 5×4 arrangement. The number of buried marbles (>50% covered by bedding material) was counted in 30 minutes by a qualified double-blinded technician (16-19).

Elevated plus maze (EPM). The EPM test was performed to characterize anxiety-like behavior in mouse offspring. The Elevated Plus Maze Package together with IR Beam Detection (Cat #: MED-ELVM-1R) was purchased from Med Associates Inc. The maze has two sets of open and closed arms, and dual sensors located at the entrance to each goal runway can differentiate between runway exploration and entrance for more accurate position detection. The experimental mouse was placed in the junction area. Movements were quantitated by infrared beams installed on each arm for 5 min and then automatically recorded by MED-PC software (Cat #: SOF-735, Med Associates). The time spent in both the open and closed arms was quantitated by a double-blinded technician. In addition, total motor activity was counted in an open-field test during a 30 min assessment (18, 20).

Open-field test (OFT). The mice were tested in an open-field arena using the Open Field Starter Package for mouse (Med Associates Inc.). The open-field arena consists of fixed position walls mounted to the system base. Animal movement is tracked using three of 16 beam IR arrays. IR beams are located on both the X and Y axes for positional tracking and the Z axis for rearing detection. The animals were placed in the center of an open field, and exploration was assessed for 30 min. The dimensions of the arena were 40 cm × 40 cm, of which the peripheral 10 cm were considered the peripheral zone and the central 20 cm were considered the central zone (18).

New object recognition (NOR) test. The NOR test was performed following open-field test, two identical objects were placed in the apparatus to eliminate any potential presence of unique odors. The mice were positioned approximately 10 cm away from the edge of the apparatus, facing the center at an equal distance. A camera was set up to capture and record the exploration time of the two objects, defined as when their noses were within 2 cm of the objects or when they made direct contact with the objects. The number of times and length of time the mice explored the two objects were recorded for 5 min. To evaluate the effect on memory improvement, a 1-h interval passed after the completion of 5 min of recording. After the dwell time was completed, one of the objects was replaced by another new object in the apparatus, and the experiment was repeated for 5 min using the above experimental method. The exploration time of the mice was observed and recorded (“N” for the novel object and “O” for the old object). The NOR test was quantified as a discrimination index N/(N+O) (21).

Three-chambered social test. 7-8 week old mice were employed for measurement of sociability and preference for social novelty. Target subjects, including Stranger 1 and Stranger 2, were placed inside the wire cages for 3 days before the beginning of testing, and the test mice were placed in the testing room for at least 45 min before the start of behavioral tests. For the sociability test, the test mouse was placed in the middle chamber and left to habituate for 5 min, after which an unfamiliar Stranger 1 mouse was introduced into a wire cage in one of the side-chambers and an empty wire cage on the other side-chamber. The test mouse was allowed to explore all 3 chambers freely for 10 min. Following this, a novel Stranger 2 mouse was introduced into the previously empty wire cage and the test mouse was again left to explore freely for 10 min. All the parameters, including time spent in each chamber, number of entries into the chambers and track maps, were recorded and calculated by automated SMART software (3, 22).

**Isolation of brain tissues**. The brain tissues were isolated from experimental mice for further biological assays. The experimental mice were deeply anesthetized through free breathing of isoflurane vapor (> 5%). The whole blood was then withdrawn by heart puncture for PBMC isolation and the mouse was perfused transcardially by 20 ml cold perfusion solution for 5 min. The skull was cut using a pair of small surgical scissors and the brain was carefully freed from the skull before being transferred to a petri dish (60 mm×15 mm) that was filled with ice-cold DPBS solution. The targeted brain regions, including the amygdala, hypothalamus (PVN area) and hippocampus, were dissected under the surgical microscope under the referred location from the atlas outlined in *The Mouse Brain in Stereotaxic Coordinates (3rd Edition)*. A separate petri dish was prepared for each of the target regions. The whole dissection process was carried out in the span of no more than one hour. The dissected tissues were then frozen at -80°C for either immediate use or later biological assays (23, 24).

**Isolation and characterization of hematopoietic stem cells (HSC)**. The HSC preparation procedure is a minor modification from previously described method (25). In brief, the whole bone marrow cells were collected from tibias in treated mice. Bone marrow cells were stained with antibodies for the identification of HSC (c-Kit^+^/Sca1^+^/Lineage^-^), and the following antibodies were used: c-Kit-PE (#12-1171-82), Sca-1-FITC (#11-5981-82), and anti-Lineage Antibody Cocktail comprises a mixture of PE-Cy5-conjugated antibodies, including anti-B220, anti-CD4, anti-CD8, anti-Gr-1, anti-Mac-1, and anti-TER119 (from eBioscience). For HSC sorting, the debris, dead and clumped cells were firstly removed to obtain the single and viable cells, then the Sca-1 positive, c-Kit positive and Lineage negative cell population were isolated by HSC sorting, and the FACS analysis was performed on BD FACSMelody™ Cell Sorter (26).

**Monitoring of the vascular function**. The vessel tension for carotid artery in mice was measured in the Multi Wire Myograph System Model 620M (from Danish Myo Technology, Denmark). The three 3-mm aortic rings from each animal were quickly excised and placed in a Krebs bicarbonate buffer (118 mmol/L NaCl, 4.7mmol/L KCl, 25 mmol/L NaHCO3,1.2 mmol/L KH2PO4, 1.2 mmol/L MgSO4, 2.5 mmol/L CaCl2, and 5 mmol/L glucose), and the adhering tissue and fat were removed. Each ring was positioned between two 40-μm stainless steel wires in an 8-ml organ myograph chamber (DMT 620M), filled with Krebs bicarbonate buffer, maintained at 37±0.5°C and aerated with 95% O2 plus 5% CO2 (pH = 7.4). At the beginning of the experiment, each vessel ring was stretched to its optimal resting tension and allowed to equilibrate for 1h. To study vasodilator responses, the acetylcholine (Ach,10−10-10–4mol/l) induced vasodilation was assessed in aortas preconstricted with phenylephrine (10–5mol/l) at a level corresponding at least to the maximal response to potassium (100mmol/l KCl). The dose-response relaxation was measured for cumulative increments of acetylcholine at 1min intervals, and the Ach-induced change in tension was expressed as the percentage of the initial contraction induced by phenylephrine (6, 13).

**Radiotelemetric blood pressure monitoring in vivo**. Blood pressure was measured in conscious mice with the radiotelemetry technique described previously. Briefly, the mice were anaesthetized by intraperitoneal injection of 100mg/kg ketamine/16mg/kg xylazine, a catheter (PE10 tubing) and the telemetry transmitter unit (TA11PA-C10, Data Sciences International (DSI)) was implanted in the left carotid artery, while the radiotransmitter was placed in a subcutaneous pouch along the flank. Mice were treated with analgesics for 3 days (buprenorphine, 0.1 mg/kg) to relieve the pain and were allowed to recover for 7 days after surgery to regain their normal circadian rhythms before blood pressure measurements. While the blood pressure was being monitored, the mice were housed in a quiet room in individual cages placed above the telemetric receivers with an output to a computer. Blood pressure was measured for 5 minutes every hour, processed, and analyzed using the DataQuest ART system (DSI) (14-16).

**Immunohistochemistry (IHC).** Slides from brain tissues containing amygdala were deparaffinized in Histoclear solution (National Diagnostics) and rehydrated in a series of ethanol. Antigen was retrieved using 10mM sodium citrate buffer (pH 6.0) at 100°C for 10 min, and then treated with 0.3% H2O2. After incubation with 0.3% Triton X-100 in PBS for 15 min and blocking with normal goat serum for 30min, tissues were incubated in primary antibodies SOD2 (#sc-133134, from Santa Cruz Biotechnology) at 4°C for 12 h, and subsequently with secondary antibodies Alexa Fluor 647 (#ab150083 from Abcam). The cover slips were then mounted by antifade Mountant with DAPI (staining nuclei, in blue). The photographs were taken by using a Confocal Laser Microscope (Leica, 20x lens) and quantitated by Image J. software (3).

**Statistical analysis.** The data are presented as mean ± standard deviation (SD), with all experiments conducted at least in quadruplicate unless otherwise specified. Normality was assessed using the Shapiro-Wilk test (27). Unpaired Student’s t-tests were used to compare differences between the hypoxia and normoxia groups in the MEC in vitro study. For the remaining experiments, one-way analysis of variance (ANOVA) followed by the Tukey-Kramer test was performed to determine statistical significance. Statistical analyses were conducted using SPSS 22, with a P-value < 0.05 considered statistically significant.

REFERENCES

1. Takeshita K, Satoh M, Ii M, Silver M, Limbourg FP, Mukai Y, et al. Critical role of endothelial Notch1 signaling in postnatal angiogenesis. *Circ Res.* 2007;100(1):70-8.

2. Bodnar AG, Ouellette M, Frolkis M, Holt SE, Chiu CP, Morin GB, et al. Extension of life-span by introduction of telomerase into normal human cells. *Science.* 1998;279(5349):349-52.

3. Wang X, Lu J, Xie W, Lu X, Liang Y, Li M, et al. Maternal diabetes induces autism-like behavior by hyperglycemia-mediated persistent oxidative stress and suppression of superoxide dismutase 2. *Proc Natl Acad Sci U S A.* 2019;116(47):23743-52.

4. Ceradini DJ, Yao D, Grogan RH, Callaghan MJ, Edelstein D, Brownlee M, et al. Decreasing intracellular superoxide corrects defective ischemia-induced new vessel formation in diabetic mice. *J Biol Chem.* 2008;283(16):10930-8.

5. Ogino S, Kawasaki T, Brahmandam M, Cantor M, Kirkner GJ, Spiegelman D, et al. Precision and performance characteristics of bisulfite conversion and real-time PCR (MethyLight) for quantitative DNA methylation analysis. *J Mol Diagn.* 2006;8(2):209-17.

6. Eads CA, Danenberg KD, Kawakami K, Saltz LB, Blake C, Shibata D, et al. MethyLight: a high-throughput assay to measure DNA methylation. *Nucleic Acids Res.* 2000;28(8):E32.

7. Nosho K, Irahara N, Shima K, Kure S, Kirkner GJ, Schernhammer ES, et al. Comprehensive biostatistical analysis of CpG island methylator phenotype in colorectal cancer using a large population-based sample. *PLoS ONE.* 2008;3(11):e3698.

8. Yao D, Shi W, Gou Y, Zhou X, Yee Aw T, Zhou Y, et al. Fatty acid-mediated intracellular iron translocation: a synergistic mechanism of oxidative injury. *Free Radic Biol Med.* 2005;39(10):1385-98.

9. Zou Y, Lu Q, Zheng D, Chu Z, Liu Z, Chen H, et al. Prenatal levonorgestrel exposure induces autism-like behavior in offspring through ERbeta suppression in the amygdala. *Mol Autism.* 2017;8:46.

10. Zhang H, Li L, Chen Q, Li M, Feng J, Sun Y, et al. PGC1beta regulates multiple myeloma tumor growth through LDHA-mediated glycolytic metabolism. *Mol Oncol.* 2018;12(9):1579-95.

11. Zhang H, Li L, Li M, Huang X, Xie W, Xiang W, et al. Combination of betulinic acid and chidamide inhibits acute myeloid leukemia by suppression of the HIF1alpha pathway and generation of reactive oxygen species. *Oncotarget.* 2017;8(55):94743-58.

12. Kobayashi EH, Suzuki T, Funayama R, Nagashima T, Hayashi M, Sekine H, et al. Nrf2 suppresses macrophage inflammatory response by blocking proinflammatory cytokine transcription. *Nat Commun.* 2016;7:11624.

13. Isaacson B, Hadad T, Glasner A, Gur C, Granot Z, Bachrach G, et al. Stromal Cell-Derived Factor 1 Mediates Immune Cell Attraction upon Urinary Tract Infection. *Cell Rep.* 2017;20(1):40-7.

14. Zeng J, Liang Y, Sun R, Huang S, Wang Z, Xiao L, et al. Hematopoietic stem cell transplantation ameliorates maternal diabetes-mediated gastrointestinal symptoms and autism-like behavior in mouse offspring. *Ann N Y Acad Sci.* 2022;1512(1):98-113.

15. Romanowicz J, Guerrelli D, Dhari Z, Mulvany C, Reilly M, Swift L, et al. Chronic perinatal hypoxia delays cardiac maturation in a mouse model for cyanotic congenital heart disease. *Am J Physiol Heart Circ Physiol.* 2021;320(5):H1873-H86.

16. Bahi A. Sustained lentiviral-mediated overexpression of microRNA124a in the dentate gyrus exacerbates anxiety- and autism-like behaviors associated with neonatal isolation in rats. *Behav Brain Res.* 2016;311:298-308.

17. Bahi A. Hippocampal BDNF overexpression or microR124a silencing reduces anxiety- and autism-like behaviors in rats. *Behav Brain Res.* 2017;326:281-90.

18. Zou Y, Lu Q, Zheng D, Chu Z, Liu Z, Chen H, et al. Prenatal levonorgestrel exposure induces autism-like behavior in offspring through ERβ suppression in the amygdala. *Mol Autism.* 2017;8:46.

19. Xie W, Ge X, Li L, Yao A, Wang X, Li M, et al. Resveratrol ameliorates prenatal progestin exposure-induced autism-like behavior through ERβ activation. *Mol Autism.* 2018;9:43.

20. Hu M, Richard JE, Maliqueo M, Kokosar M, Fornes R, Benrick A, et al. Maternal testosterone exposure increases anxiety-like behavior and impacts the limbic system in the offspring. *Proc Natl Acad Sci U S A.* 2015;112(46):14348-53.

21. Kong X, Lyu W, Lin X, Lin C, Feng H, Xu L, et al. Itaconate alleviates anesthesia/surgery-induced cognitive impairment by activating a Nrf2-dependent anti-neuroinflammation and neurogenesis via gut-brain axis. *J Neuroinflammation.* 2024;21(1):104.

22. Moy SS, Nadler JJ, Perez A, Barbaro RP, Johns JM, Magnuson TR, et al. Sociability and preference for social novelty in five inbred strains: an approach to assess autistic-like behavior in mice. *Genes Brain Behav.* 2004;3(5):287-302.

23. Liu L, Zhou X, and Wu JY. Preparing Viable Hippocampal Slices from Adult Mice for the Study of Sharp Wave-ripples. *Bio Protoc.* 2020;10(19):e3771.

24. Liu L, Besson-Girard S, Ji H, Gehring K, Bulut B, Kaya T, et al. Dissociation of microdissected mouse brain tissue for artifact free single-cell RNA sequencing. *STAR Protoc.* 2021;2(2):100590.

25. Rossi L, Challen GA, Sirin O, Lin KK, and Goodell MA. Hematopoietic stem cell characterization and isolation. *Methods Mol Biol.* 2011;750:47-59.

26. Xie W, Zhou X, Hu W, Chu Z, Ruan Q, Zhang H, et al. Pterostilbene accelerates wound healing by modulating diabetes-induced estrogen receptor beta suppression in hematopoietic stem cells. *Burns Trauma.* 2021;9:tkaa045.

27. Ramezani G, Norouzi A, Arabshahi SKS, Sohrabi Z, Zazoli AZ, Saravani S, et al. Study of medical students' learning approaches and their association with academic performance and problem-solving styles. *J Educ Health Promot.* 2022;11:252.

**Table S1. Sequences of primers for the real time quantitative PCR (qPCR)**

| Gene | Species | Analysis | Forward primer (5'→3') | Reverse primer (5'→3') |
| --- | --- | --- | --- | --- |
| β-actin | Mouse | mRNA | tcttgggtatggaatcctgtg | atctccttctgcatcctgtca |
| ERβ | Mouse | mRNA | atgtgctatggccaacttctg | caagcttcctcttcagggtct |
| HIF1α | Mouse | mRNA | gcagcaggaattggaacatt | gcatgctaaatcggagggta |
| IL1β | Mouse | mRNA | gcccatcctctgtgactcat | tccattgaggtggagagctt |
| IL6 | Mouse | mRNA | ctgatgctggtgacaaccac | tttccacgatttcccagaga |
| MCP1 | Mouse | mRNA | tcctcaggtattggctggac | tggtcctgaagatcacagctt |
| SOD2 | Mouse | mRNA | ggcctacgtgaacaatctcaa | tcaggtttgtccagaaaatgg |
| SYP | Mouse | mRNA | ttcgctttcatgtggctagtt | aagtcacagggtccctcagtt |
| TNFα | Mouse | mRNA | ctcacaaaccaccaagtgga | ccttgtcccttgaagagaacc |
| VEGF | Mouse | mRNA | ccaaagccagcacatagagag | catttacacgtctgcggatct |
| ERβ | Mouse | ChIP | atgacacgcacgtaggtcaa | cccgcttccaatagactgac |

FIGURE S1

**Figure S1. Representative pictures of full blots for Western Blotting.** (a) Full blots for Figure 2g. (b) Full blots for Figure 3c. (c) Full blots for Figure 5c. (d) Full blots for Figure 8c.

FIGURE S2

**Figure S2. Hypoxia and related factors released from MEC-mediated epigenetic modifications on the ERβ promoter in amygdala neurons.** Amygdala neurons were isolated from untreated mouse on E18, the cells were infected by either SOD2 lentivirus (↑SOD2) or empty control (EMP), then incubated in the medium with 50% (v/v) of supernatant from either hypoxia (factor) or normoxia (CTL)-treated MEC cells, in the presence of either normoxia (NO) or hypoxia (HO) for 48 hours, and the above treated cells were collected for biological assays. (a) histone 4 methylation on the ERβ promoter. (b) histone acetylation on the ERβ promoter. n=4, results are expressed as mean ± SD.

FIGURE S3

**Figure S3. Prenatal hypoxia exposure triggers altered gene expression in brain tissues in offspring, while ERβ expression in endothelium partly ameliorates this effect.** Female mice were injected by either Tie2-driven ERβ expression lentivirus (Tie2-↑ERβ), Tie2-driven ERβ knock down lentivirus (Tie2-shERβ), or Tie2-driven empty lentivirus (Tie2-EMP), after pregnancy, the mice were treated either in normoxia (NO) or hypoxia (HO) condition during the period of E8-E21, after baby delivery, the offspring were fed in normoxia conditions after P8 and used for further biomedical analysis on P30. (a) mRNA levels in hypothalamus. (b) mRNA levels in hippocampus. n=4, results are expressed as mean ± SD.
